# Supplementary material for: Development and Validation of the Midwifery Interventions Classification for a Salutogenic Approach to Maternity Care: A Delphi Study
Source: Healthcare (Basel). 2024 Nov 8;12(22):2228. doi: 10.3390/healthcare12222228 (PMC11594468; doi:10.3390/healthcare12222228)
Supplement: Supplementary file 1 [file healthcare-12-02228-s001.zip › Table S1.pdf]

Table S1. List of 165 midwifery interventions presented to participants in Round 1 of the Delphi survey
***In the Italian language, this list of midwifery interventions was presented in alphabetical order**

Category: Direct Midwifery Care

N.	Midwifery intervention	Definition
1	Welcome upon arrival at the facility	Welcome the birthing person upon arrival at a health or social care facility.
2	Bottle feeding	Prepare and administer a bottle feed of expressed milk or newborn infant formula.
3	Cup feeding	Prepare and administer a cup feed of expressed milk or infant formula.
4	Management of newborn nutrition	Ensure adequate nutrition for the newborn.
5	Management of nutrition in pregnancy/childbirth/postpartum	Ensure adequate nutrition in the pregnancy/birth/postpartum phases.
6	Facilitating breastfeeding initiation	Support with breastfeeding initiation.
7	Facilitate breastfeeding continuation	Support with breastfeeding continuation.
8	Active listening	Pay attention to and attach meaning to verbal and nonverbal communication by the parent(s) and the newborn.
9	First stage of labour care	Care and support for the birthing person from the beginning of labour until complete cervical dilation.
10	Second stage of labour care	Care from complete cervical dilation until the birth of the newborn.
11	Third stage of labour care	Care between the birth of the newborn and the birth of the placenta.
12	Fourth stage of labour care	Care in the first 2 hours after giving birth.
13	Supporting newborn adaptation	Support the newborn in the transition phase from birth to extrauterine life and the subsequent stabilization phase.
14	Prodromal labour care	Support the birthing person from the beginning of the perception of strong uterine contractions until the start of labour.
15	Birth care	Care during childbirth.
16	Antenatal care	Care during pregnancy.
17	Postnatal care	Care in the 6 weeks following childbirth.
18	Management of cardiotocographic changes	Resolve changes in fetal heart rate (FHR) and/or uterine contractions (UC) detected by cardiotocography.
19	Cord clamping	Clamp the umbilical cord at an appropriate timing.
20	Goals sharing	Work with the pregnant/postpartum person to identify expected health outcomes, prioritise among them, and develop an action plan.
21	Telephone consultations	Enable the person to express concerns through listening and support over the phone, and offering information or advice as appropriate.
22	Skin to skin	Lay the newborn directly on the birthing person's bare chest after birth, covered in a warm blanket, for at least an hour or until after the first feed. Skin-to-skin contact can also occur whenever a baby needs comforting or calming and can help boost milk supply.
23	Negotiation of care with birthing person	Negotiate a deal that will support the birthing person to change a specific behaviour.
24	Counselling	Use an interactive supportive process focused on the birthing person's needs, problems, or feelings to improve or support coping, problem-solving, and interpersonal relationships.
25	Counselling pregnant people on respectful maternity care	Counsel pregnant people on their right to respectful perinatal care.
26	Sexual health counselling	Counsel the birthing person on sexual health during pregnancy and the postnatal period.
27	Newborn care counselling	Counsel the birthing person and their family on nutrition and care of the newborn.
28	Breastfeeding counselling	Counsel the birthing person on breastfeeding, following the 10 steps to successful breastfeeding by WHO/UNICEF.
29	Healthy behaviour counselling	Counsel the birthing person and family on health promotion behaviours.
30	Prenatal diagnosis counselling	Counsel the birthing person and family on screening and prenatal diagnosis methods.

31	Newborn bowel movements counselling	Counsel the birthing person and family on the physiology of newborn bowel movements.
32	Counselling the birthing person on their own bowel movements	Counsel the birthing person on healthy bowel habits.
33	Newborn urinary counselling	Counsel the birthing person and family on the physiology of newborn urinary elimination.
34	Counselling the birthing person on their own urinary elimination	Counsel the birthing person on healthy urinary elimination habits.
35	Newborn nutrition counselling	Counsel the birthing person and family on newborn nutrition, following the 10 steps to successful breastfeeding by WHO/UNICEF.
36	Nutrition counselling for the birthing person	Counsel the birthing person on their own nutrition and advise on the need to make dietary changes.
37	Family planning counselling	Counsel the birthing person and family on contraception and family planning.
38	Signs and symptoms counselling	Counsel the birthing person on how to recognise signs and symptoms for which they should consult a healthcare professional.
39	Counselling on newborn safety	Counsel the birthing person and family on keeping the newborn safe.
40	Umbilical cord care	Promote healing of the newborn umbilical stump and prevent complications.
41	Perineal care	Prevent perineal tearing and relieve perineal discomfort during childbirth.
42	Perineal tear care	Promote the healing of perineal tears and prevent tear complications.
43	Point-of-Care Ultrasound (POCUS)	During a midwifery consultation, carry out an ultrasound examination to gain additional clinical information.
44	Management of newborn bowel movements	Monitor and manage the newborn's healthy bowel movements.
45	Management of the birthing person's bowel movements	Monitor and support the birthing person's healthy bowel movements.
46	Management of the newborn's micturition	Monitor the newborn's passing of urine.
47	Management of the birthing person's micturition	Monitor the birthing person's passing of urine.
48	Newborn physical examination	Assess the newborn's health through a physical examination.
49	Physical examination of the birthing person	Assess the birthing person's health through a physical examination.
50	Learning process facilitation	Promote the birthing person’s ability to process and understand information.
51	Telephone follow-up	By telephone, provide the results of examinations, evaluate the birthing person’s response, and discuss potential problems resulting from previous treatments or tests.
52	Pain management	Eliminate or reduce pain to a level acceptable to the birthing person and newborn.
53	Allergy management	Identify, treat, and prevent allergic reactions to food, drugs, latex, insect bites, contrast media, blood, and other substances.
54	Newborn prophylaxis	Carry out recommended prophylaxis interventions recommended by the guidelines in the first hours of newborn life.
55	Guidelines on how to prevent critical situations	Prepare the birthing person for a foreseeable development or situational crisis.
56	Identification of the birthing person and/or newborn	Verify the identity of the birthing person and/or newborn.
57	Hydrotherapy/ immersion in water	Use water in labour and/or childbirth (i.e., shower or birthing pool) to enhance coping and pain management.
58	Newborn hygiene management	Ensure adequate newborn hygiene.
59	Hygiene management for the birthing person	Ensure adequate hygiene for the birthing person.
60	Intravenous cannulation	Insert an intravenous cannula into a peripheral vein to administer fluids, blood, or medication.
61	Massage	Stimulate the skin and underlying tissues through a variable degree of manual pressure to reduce pain, facilitate relaxation, and improve circulation for the birthing person and newborn.
62	Coping enhancement	Facilitate cognitive processing and the adoption of behaviours for managing factors perceived as stressful. These could include changes and threats that interfere with the satisfaction of needs and the performance of roles.
63	Promotion of health literacy	Support the birthing person and family to acquire, process, and understand information related to health and disease.
64	Promotion of self-efficacy	Strengthen the birthing person’s confidence in their ability to engage in healthy behaviours.

65	Boosting self-confidence	Counsel the birthing person on valuing and trusting themselves.
66	Enhancing willingness to learn	Enhance the birthing person's capacity and willingness to receive information.
67	Facilitating social connections and support	Facilitate the birthing person's ability to interact with other people.
68	Monitoring of vital signs	Systematically collect and analyze data on cardiovascular and respiratory functions and body temperature to identify and prevent complications in the birthing person and newborn.
69	Early postnatal observation	Assess signs of physical and emotional well-being in the 2 hours after birth, in the birthing person and newborn.
70	Promotion of self-awareness	Support the birthing person in exploring and understanding their thoughts, feelings, motivations, and behaviours.
71	Role empowerment	To help the birthing person and/or the family to improve their relationship by clarifying and integrating specific role-playing behaviours.
72	Capillary blood sampling	Take a blood sample from a peripheral site, such as the heel, finger, or other transcutaneous site, from the birthing person and newborn.
73	Venous blood sampling	Take a venous blood sample from a peripheral vein in the birthing person and newborn.
74	Antenatal class	Provide information and support to facilitate childbirth and promote the woman's ability to develop and perform the parent role.
75	Presence	Remain close to the birthing person and/or the newborn, both physically and psychologically, whenever needed.
76	Postpartum haemorrhage prevention	Implement post-partum haemorrhage-prevention measures following the adopted guidelines.
77	Facilitation of free movement in labour	Promote freedom of movement and changing positions during labour.
78	Parental role promotion	Provide information on parenting, signpost, and support services available to families.
79	Promotion of parental-infant bonding	Support the development of a stable bond and an emotional relationship between child and parents.
80	Empowerment promotion	Promote the birthing person's skills activation related to pregnancy, childbirth, and postnatale period.
81	Promotion of self-care	Support the birthing person's willingness and ability to take care of themselves physically and emotionally.
82	Promotion of free movement and positions during labour and birth	Promote the birthing person's choice on what positions to use during birth.
83	Body temperature regulation	Ensure the birthing person's and newborn's body temperature are maintained within a normal range.
84	Administration of analgesia	Use medication to reduce or eliminate pain.
85	Administration of medication	Prepare, administer, and assess the effectiveness of prescription and over-the-counter medication.
86	Maternal-fetal monitoring in labour	Monitor and document maternal-fetal well-being and the progression of labour over time.
87	Monitoring of term pregnancy	Acquire, interpret, and synthesize maternal and fetal well-being consistently and meaningfully for treatment, monitoring, or hospitalization.
88	Telehealth	Acquire meaningful data on the birthing person and/or the newborn from a remote location through electronic tools (telephone, videoconference, e-mail), interpret and synthesize them to support appropriate clinical care.
89	Caregiver support	Provide the necessary information and support to facilitate the provision of primary care to the birthing person and the newborn by someone who is not a health professional.
90	Decision-making support	Provide information and support to the birthing person and family when making decisions about healthcare.
91	Emotional support	Offer reassurance, acceptance and encouragement to the birthing person and/or the newborn in times of stress.
92	Support to manage behaviour	Support the birthing person in managing her behaviour by directing her towards health promotion.
93	Support for behaviour change	Promote behavioural change.
94	Perineal repair	Bring the edges of a perineal tear closer, using sterile suture material and a needle.

95	Obstetric triage	Briefly, thoroughly, and systematically assess the maternal-fetal well-being of a birthing person who needs assistance to determine the priority for a complete evaluation.
96	Telephone triage	Via telephone, determine the nature and urgency of one or more reported issues and provide advice to birthing people.
97	Emotional well-being assessment	Assess the birthing person's emotional well-being during pregnancy, birth, and the postnatal period.
98	Fetal well-being assessment: FHR	Assess fetal well-being through auscultation and/or fetal heart rate (FHR) recording.
99	Fetal well-being assessment: FM	Assess fetal well-being through fetal movement (FM) assessment.
100	Assessment of uterine contractions	Assess uterine contractions via palpation and/or tocography.
101	Guided visualization	Direct a person's imagination in a targeted manner to achieve a particular state, result. or action, or to shift their attention away from unwanted sensations.

Category: Indirect Midwifery Care

N.	Midwifery Intervention	Definition
102	Care at home	Provide care to the birthing person and/or the newborn in their own home.
103	Case managament	Coordinate the care and protection of women in different contexts to improve health care quality, ensure continuity of care, and achieve the desired results by mobilising necessary and sufficient resources.
104	Caregiver engagement	Involve and engage with the birthing's person's chosen supporter or caregiver during pregnancy, labour, childbirth, and postnatal period.
105	Team cooperation	Collaborate and work in synergy with the members of the clinical team to ensure quality care.
106	Midwifery clinical handover	Share essential and sufficient clinical information with another midwifery team upon shift change or transfer to another facility.
107	Midwifery clinical consultation	By mobilising expert knowledge, support birthing people, newborns, and the community to achieve their identified goals related to health and well-being.
108	Multidisciplinary clinical consultation	Plan and evaluate care for the birthing person and/or the newborn in collaboration with health professionals from other disciplines.
109	Midwife-led continuity of carer	Ensure that an individual midwife, or her buddy/backup, provide the majority of care to a birthing person throughout pregnancy, childbirth, and the postnatal period.
110	Emergency trolley check	Systematically and regularly check and re-stock the emergency and crash trolleys.
111	Infection prevention and control	Prevent the transmission of infectious agents.
112	Clinical record keeping	Document data related to the birthing person and newborn in their health record (midwifery or integrated health record, printed or digital).
113	Partograph	Record data relating to labour progression and birth in the partograph.
114	Facilitating visits by family and friends	Follow the birthing person's wishes in enabling and supporting their friends and family to visit.
115	Training healthcare professionals on respectful perinatal care	Implement training courses for healthcare personnel that promote knowledge and increase skills on respectful perinatal care and related best practices.
116	Management of supplies	Acquire and appropriately manage supplies necessary for clinical care provision.
117	Management of laboratory samples	Take, prepare and appropriately store samples for laboratory testing.
118	Management of acuity codes	Coordinate emergency measures for life support.
119	Medication management	Promote safe and effective use of prescribed and over-the-counter medications.
120	Management of the environment	Arrange the physical environment surrounding the birthing person and/or the newborn in such a way to promote health, sensory satisfaction, and psychological well-being.
121	Environmental well-being management	Organize the environment around the birthing person and the newborn to promote respectful maternity care and an optimal state of well-being.
122	Environmental safety management	Monitor and arrange the physical environment to promote safety.
123	Management of technical equipment and devices	Use technical equipment and devices appropriately to monitor or support the vital functions of the birthing person and/or newborn.

124	Provide guidance on navigating the healthcare service	Ensure that the birthing person and the family are able to identify and access health services appropriate to their condition.
125	Risk identification	Analyse potential risk factors for the birthing person, the newborn and the family, determine health risks, and prioritise risk-reduction strategies.
126	Implementation of healthcare quality improvement programs	Implement quality improvement programs and/or regular audits to ensure respectful perinatal care.
127	Implementation of regulations/recommendations and guidelines for respectful perinatal care	Implement standardized checklists and review hospital or regional care pathways to ensure respectful perinatal care.
128	Incident reporting	Document any event that occurred during the care process, which was inconsistent with expected outcomes for the birthing person and/or newborn, or with the routine functioning of the health facility.
129	Interpretation of laboratory data	Critically analyze laboratory data relative to the birthing person and newborn and use it to support clinical decision-making.
130	Cultural mediation	Intentionally use strategies based on cultural competence to overcome a gap or to mediate between the birthing person's culture and the health system.
131	Enhancing interdisciplinary collaboration	Strive to achieve cooperation between healthcare professionals from different disciplines and backgrounds.
132	Quality monitoring	Systematically collect and analyse information related to the health organisation's quality indicators.
133	Personalized care	Plan and deliver midwifery care focused on individuals' history, needs, and preferences.
134	Care planning	Plan midwifery care in collaboration with the birthing person.
135	Discharge planning	Plan the discharge home or the transfer of the birthing person and/or newborn from one level of care to another within a health facility, or to another facility.
136	Latex use precautions	Take precautions to reduce the risk of systemic reaction to latex.
137	Diagnostic test prescription	Prescribe a diagnostic test to identify or monitor a health problem.
138	Non-pharmacological treatment prescription	Prescribe non-pharmacological treatment for a health problem or to support body functions.
139	Prevention of falls	Take specific precautions to avoid falls.
140	Advocate for respectful perinatal care	Promote and ensure respectful perinatal care.
141	Rooming-in	Ensure the birthing person and the newborn are able to stay together for 24 hours a day, while they are in the facility.
142	Communication of healthcare information	Provide information about the birthing person and/or the newborn to other healthcare professionals.
143	Supervision of healthcare personnel	Facilitate the provision of high-quality care activities by other healthcare personnel, through supervision.
144	Support for those providing respectful perinatal care	Implement actions and awards to support those who provide respectful perinatal care.
145	Healthcare personnel development	Monitor, maintain, and further develop staff skills.
146	Transfer within the facility	Transfer the birthing person and/or the newborn from one area to another of the same healthcare facility.
147	Protecting the rights of birthing people and newborns	Protect the rights of the birthing person, the newborn, and their family.
148	Orientation of healthcare personnel	Support a newly appointed or transferred team member by orienting them to a new clinical area.
149	Mentorship and supervision of students	Mentor, supervise, and support student learning experiences.
150	Assessment of new equipment	Determine the effectiveness of new equipment.
151	Risk assessment for the fetus/newborn	Analyse potential obstetric risk factors for the fetus/newborn, determine health risks, and prioritise risk-reduction strategies.
152	Risk assessment for the birthing person	Analyse potential obstetric risk factors for the birthing person, determine health risks, and prioritise risk-reduction strategies.

Category: Community Midwifery Care

N.	Midwifery Intervention	Definition

153	Implementation of social accountability for respectful perinatal care	Improve the responsiveness and accountability of the service to the community, relative to respectful perinatal care.
154	Engagement of community members with respectful perinatal care	Educate community members on the importance of respectful perinatal care and involve them as community supporters.
155	Health education	Develop and provide information and experiences to facilitate the voluntary adoption of healthy behaviours.
156	Budget management	Contribute to the management of funding streams and to decisions around budget allocation, to support the development and continuity of programmes and services.
157	Management of vaccinations	Monitor immune status and facilitate access to vaccinations to prevent communicable diseases.
158	Monitoring of health policy	Keep track of and exert influence over the development of national and local regulations, norms, and standards that affect systems, midwifery practice, and ultimately, the health and well-being of the population.
159	Promotion of physical activity/exercise	Promote regular physical activity to maintain and enhance fitness and health.
160	Promotion of cord blood donation	Facilitate cord blood donation for solidaritistic purposes.
161	Promotion of physiological processes during labour and birth	Provide care that facilitates and enhances physiological processes during childbirth.
162	Data collection for research purposes	Contribute to data collection for research activities seeking to expand the evidence base related to midwifery care.
163	Screening	Detect risk factors or health problems using medical history, physical examination, and other diagnostic investigations.
164	Promoting health awareness at community level	Support community members in identifying health concerns, activating resources, and implementing solutions.
165	Development of health programmes	Plan, implement, and evaluate a coordinated set of activities to improve well-being or prevent, reduce, or eliminate one or more health problems in birthing people, families, or the community.
