# Supplementary material for: Development and Validation of the Midwifery Interventions Classification for a Salutogenic Approach to Maternity Care: A Delphi Study
Source: Healthcare (Basel). 2024 Nov 8;12(22):2228. doi: 10.3390/healthcare12222228 (PMC11594468; doi:10.3390/healthcare12222228)
Supplement: Supplementary file 1 [file healthcare-12-02228-s001.zip › Table S2.pdf]

Table S2: results of ANOVA mixed

Item	Category *p-value*	Time *p-value*	Interaction *p-value*
Item1	0.799597677	0.780526553	0.934193535
Item2	5.21955E-06	0.793118725	0.921268341
Item3	0.000210472	0.782150149	0.746433035
Item4	0.120178814	0.740490395	0.796468163
Item5	1.14042E-05	0.314050698	0.528463181
Item6	2.62103E-05	0.683570157	0.779353159
Item7	0.000193603	0.969388618	0.968724941
Item8	0.030392729	0.946259219	0.531303563
Item9	1.36811E-07	0.467271531	0.393321859
Item10	0.001426866	0.647195051	0.616053237
Item11	0.270247702	0.674003545	0.68633584
Item12	0.777625823	0.797231338	0.814284374
Item13	0.124307508	0.930649307	0.838722843
Item14	0.042143616	0.364464301	0.500185899
Item15	0.971300835	0.997626536	0.985244245
Item16	2.36053E-14	0.594342387	0.372986928
Item17	3.36359E-12	0.826191058	0.822778991
Item18	0.010866379	0.785907547	0.755481129
Item19	0.19758017	0.633880715	0.657886544
Item20	1.45923E-07	0.588964928	0.345423058
Item21	0.047632625	0.611535214	0.67178578
Item22	0.000116479	0.978574381	0.865969377
Item23	0.359618535	0.943755177	0.948201988
Item24	1.90006E-07	0.64280497	0.522529416
Item25	0.001822585	0.693899823	0.707733899
Item26	0.00025606	0.902550678	0.398318729
Item27	0.001078036	0.614620499	0.344948386
Item28	0.001144547	0.763694196	0.389234758
Item29	0.000275415	0.71468592	0.550542767
Item30	0.520351457	0.871273433	0.951375818
Item31	0.035137448	0.769758917	0.782999401
Item32	0.00529192	0.9656678	0.968619442
Item33	0.036984465	0.862378894	0.848295475
Item34	0.002969544	0.882232996	0.977834435
Item35	0.292072083	0.701158286	0.683644558
Item36	7.29904E-07	0.811070261	0.618063499
Item37	8.87217E-06	0.889968658	0.470457499
Item38	1.00017E-05	0.660723849	0.801321015
Item39	0.000607229	0.659992306	0.458472397
Item40	0.001416336	0.775034347	0.869875722
Item41	4.59517E-06	0.813771071	0.696533521

Item42	0.017036327	0.987118053	0.816744671
Item43	0.375690327	0.815328275	0.88532802
Item44	0.005621873	0.651206406	0.921480459
Item45	0.000222667	0.766125808	0.956326242
Item46	0.007809205	0.656607671	0.834839026
Item47	5.43612E-05	0.725563968	0.944069238
Item48	0.006769363	0.767280544	0.849372181
Item49	0.000861891	0.845699705	0.95722107
Item50	0.789243862	0.966267784	0.935219538
Item51	0.000214119	0.950016583	0.55304733
Item52	0.07872231	0.993089994	0.618140623
Item53	0.52362299	0.871543128	0.944637959
Item54	0.372335572	0.769250207	0.835337542
Item55	0.542017412	0.771329331	0.869190941
Item56	1.09374E-10	0.875261743	0.753242409
Item57	0.030810573	0.714483507	0.732999006
Item58	0.132117871	0.839381266	0.354652422
Item59	0.003955285	0.826448178	0.77185829
Item60	0.000325498	0.795352483	0.729828936
Item61	0.03622926	0.888199523	0.742257221
Item62	0.024510197	0.697870798	0.905561941
Item63	0.12933427	0.943377893	0.918374547
Item64	0.050217125	0.913156978	0.832570101
Item65	0.10973301	0.807497651	0.949224635
Item66	0.981508009	0.719692046	0.934979792
Item67	0.375507453	0.678690196	0.992267663
Item68	0.017508367	0.405958965	0.367693877
Item69	0.033495922	0.572395794	0.594620682
Item70	0.90200631	0.991544445	0.974389937
Item71	0.18016797	0.889648974	0.978602483
Item72	0.085016085	0.825901776	0.731638096
Item73	0.146471817	0.949196236	0.916970295
Item74	0.537056846	0.859299076	0.767981349
Item75	0.218001866	0.957756574	0.95712053
Item76	0.831904113	0.788830498	0.906424028
Item77	0.00170933	0.870130366	0.641986216
Item78	0.169895397	0.958632018	0.773726094
Item79	0.008906097	0.767967279	0.722999283
Item80	0.007771318	0.42534761	0.845598253
Item81	0.008895841	0.821260105	0.716246897
Item82	0.003393635	0.820227395	0.717387103
Item83	0.000323795	0.941923711	0.938652307
Item84	0.082763291	0.974777845	0.724810961
Item85	0.533657354	0.887439002	0.905937634

Item86	0.497918553	0.620177303	0.831915967
Item87	0.14189501	0.558048359	0.652178422
Item88	0.00032843	0.969163074	0.744440134
Item89	0.182394155	0.870014236	0.937232588
Item90	0.004690482	0.633733154	0.793566902
Item91	0.086465896	0.866943689	0.818455104
Item92	0.180118538	0.710270913	0.990663965
Item93	0.300248587	0.647312572	0.991522547
Item94	0.614844849	0.996524655	0.94640936
Item95	0.198531574	0.914278689	0.782176692
Item96	0.038793259	0.971772791	0.492836313
Item97	0.004206085	0.717655825	0.597446555
Item98	0.034527611	0.643350021	0.625773366
Item99	0.00501797	0.753260279	0.643952601
Item100	1.08483E-07	0.979761841	0.681409091
Item101	0.246102318	0.714645928	0.836570343
Item102	0.013257883	0.396583731	0.941922133
Item103	0.023995703	0.952446574	0.99911885
Item104	0.154626378	0.904963754	0.950396209
Item105	1.3964E-05	0.923668954	0.988757397
Item106	0.000621027	0.865068316	0.923955712
Item107	0.113995352	0.873396384	0.937904475
Item108	0.006030855	0.876197138	0.981580877
Item109	0.00180193	0.873917235	0.92798482
Item110	0.150207276	0.97679531	0.803964791
Item111	0.523632164	0.907866096	0.949473765
Item112	0.181928048	0.561304965	0.891624633
Item113	0.306268611	0.979148436	0.979318376
Item114	0.067325418	0.670529205	0.932456852
Item115	0.246873025	0.710877717	0.783274306
Item116	0.145181701	0.467627453	0.709083326
Item117	0.610546197	0.532641069	0.813715181
Item118	0.910333179	0.73797639	0.847710159
Item119	0.796462968	0.569091783	0.796250577
Item120	0.101714499	0.481290565	0.583872976
Item121	0.20977495	0.581009765	0.537109716
Item122	0.276808901	0.672735602	0.761151868
Item123	0.166836199	0.632056011	0.547023292
Item124	0.12671773	0.684987855	0.86861811
Item125	0.002782872	0.575539779	0.372075968
Item126	0.182555854	0.857214701	0.6005648
Item127	0.039676665	0.952494356	0.777873609
Item128	0.383798122	0.724964693	0.595644546
Item129	0.015967724	0.733693828	0.741410782

Item130	0.037342248	0.873812684	0.41629669
Item131	0.267272013	0.73775626	0.669093665
Item132	0.042311431	0.935789953	0.951442655
Item133	0.001975569	0.923041558	0.709924093
Item134	0.002395528	0.843811783	0.424852575
Item135	0.002318809	0.840069105	0.977379167
Item136	0.000607893	0.580624287	0.773039357
Item137	0.335955164	0.515356344	0.587228291
Item138	0.05025775	0.797627124	0.671899171
Item139	0.17484291	0.65714423	0.707134749
Item140	0.441038215	0.797672568	0.865213266
Item141	1.56229E-09	0.719732948	0.982422101
Item142	0.10749041	0.958902577	0.872414979
Item143	0.560214387	0.7249716	0.834677047
Item144	0.306938488	0.684074745	0.650559032
Item145	0.099616735	0.821213678	0.56423771
Item146	0.217191393	0.447942566	0.894367314
Item147	0.955383677	0.846720838	0.853210917
Item148	0.151684386	0.805783128	0.909412806
Item149	0.012419629	0.555269452	0.463214052
Item150	0.738826678	0.752812671	0.723990359
Item151	0.104038337	0.900810145	0.608955134
Item152	0.099406372	0.93108385	0.517839013
Item153	0.079863904	0.745735823	0.754004449
Item154	0.017626137	0.754303118	0.821525567
Item155	0.152889477	0.697106669	0.909209655
Item156	0.044176115	0.58457713	0.635470425
Item157	0.026908569	0.974049115	0.900565465
Item158	0.001375262	0.963852944	0.909668825
Item159	0.021924796	0.692754867	0.673363324
Item160	0.045908631	0.965040347	0.6296359
Item161	0.735853513	0.839102502	0.932871732
Item162	0.033204368	0.618066877	0.803650293
Item163	0.078283634	0.973328424	0.953606229
Item164	0.005529999	0.853337201	0.945936648
Item165	0.110323172	0.752477691	0.993592883

The p-value is considered significant if it is less than 0.05.
The comparison was conducted only for items assessed in both rounds, excluding items that were added or modified in the second round only.

Descriptive statistics of ANOVA mixed

Category	Item_Round1	mean_Round1	sd_Round1	Item_Round2	mean_Round2	sd_Round2
MIDWIFES	Round1_item2	**5.231707317**	2.062593241	Round2_item2	5.362318841	2.007127114
RESEARCHER	Round1_item2	**6.083333333**	2.314316445	Round2_item2	6.181818182	2.400757456
USER	Round1_item2	**7.037037037**	1.742709682	Round2_item2	6.888888889	1.490711985
MIDWIFES	Round1_item3	**5.93902439**	2.116061803	Round2_item3	**6.15942029**	2.076643137
RESEARCHER	Round1_item3	**6.5**	2.195035721	Round2_item3	**6.454545455**	2.296241989
USER	Round1_item3	**4.636363636**	2.71758742	Round2_item3	**4.25**	2.352303835
MIDWIFES	Round1_item5	7.524390244	1.353783995	Round2_item5	**7.449275362**	1.419778689
RESEARCHER	Round1_item5	8.416666667	0.900336637	Round2_item5	**8.363636364**	0.924416278
USER	Round1_item5	7.033333333	1.401559066	Round2_item5	**6.476190476**	1.167006753
MIDWIFES	Round1_item6	8.695121951	0.731800155	Round2_item6	8.782608696	0.565414091
RESEARCHER	Round1_item6	8.916666667	0.288675135	Round2_item6	8.909090909	0.301511345
USER	Round1_item6	8.258064516	1.031753909	Round2_item6	8.181818182	1.006472559
MIDWIFES	Round1_item7	8.475609756	1.199028514	Round2_item7	8.507246377	1.232204963
RESEARCHER	Round1_item7	8.833333333	0.389249472	Round2_item7	8.818181818	0.404519917
USER	Round1_item7	7.833333333	1.14721051	Round2_item7	7.772727273	1.151885336
MIDWIFES	Round1_item8	8.512195122	0.82017617	Round2_item8	8.594202899	0.773054305
RESEARCHER	Round1_item8	8.833333333	0.389249472	Round2_item8	8.818181818	0.404519917
USER	Round1_item8	8.4	0.855005545	Round2_item8	8.19047619	0.92838826
MIDWIFES	Round1_item9	8.56097561	1.16637627	Round2_item9	**8.565217391**	1.218464087
RESEARCHER	Round1_item9	8.75	0.866025404	Round2_item9	**8.727272727**	0.904534034
USER	Round1_item9	7.533333333	1.995397002	Round2_item9	**6.904761905**	2.256208366
MIDWIFES	Round1_item10	8.695121951	1.107650437	Round2_item10	8.710144928	1.164441917
RESEARCHER	Round1_item10	8.75	0.866025404	Round2_item10	8.727272727	0.904534034
USER	Round1_item10	8.2	1.095445115	Round2_item10	7.857142857	1.195228609
MIDWIFES	Round1_item14	7.585365854	1.706568425	Round2_item14	**7.507246377**	1.803780249
RESEARCHER	Round1_item14	7.666666667	2.059714602	Round2_item14	**7.636363636**	2.15743956
USER	Round1_item14	7.133333333	2.1772069	Round2_item14	**6.333333333**	2.415229458
MIDWIFES	Round1_item16	8.56097561	0.721753027	Round2_item16	8.594202899	0.671233294
RESEARCHER	Round1_item16	8.833333333	0.577350269	Round2_item16	8.818181818	0.603022689
USER	Round1_item16	7.533333333	1.479359911	Round2_item16	7.142857143	1.458962449
MIDWIFES	Round1_item17	8.609756098	0.732930825	Round2_item17	8.68115942	0.675032758
RESEARCHER	Round1_item17	8.75	0.621581561	Round2_item17	8.727272727	0.646669791
USER	Round1_item17	7.666666667	1.268540659	Round2_item17	7.571428571	1.075705748
MIDWIFES	Round1_item18	8.451219512	1.297218668	Round2_item18	8.594202899	1.287026219
RESEARCHER	Round1_item18	8	2.215646838	Round2_item18	7.909090909	2.30019762
USER	Round1_item18	7.909090909	1.23091491	Round2_item18	7.6875	1.25
MIDWIFES	Round1_item20	8.134146341	1.074533434	Round2_item20	**8.15942029**	1.009122922
RESEARCHER	Round1_item20	8.5	0.797724035	Round2_item20	**8.636363636**	0.674199862
USER	Round1_item20	7.148148148	2.265183024	Round2_item20	**6.5**	2.625783091
MIDWIFES	Round1_item21	**6.317073171**	1.632070931	Round2_item21	**6.31884058**	1.658248133
RESEARCHER	Round1_item21	**7.25**	1.712255291	Round2_item21	**7.181818182**	1.778661397

USER	Round1_item21	**6.766666667**	1.675036455	Round2_item21	**6.285714286**	1.585649934
MIDWIFES	Round1_item22	8.329268293	1.1117207	Round2_item22	8.376811594	1.030440358
RESEARCHER	Round1_item22	9	0	Round2_item22	9	0
USER	Round1_item22	7.838709677	1.61445087	Round2_item22	7.681818182	1.644221154
MIDWIFES	Round1_item24	8.207317073	0.912705987	Round2_item24	8.231884058	0.909830746
RESEARCHER	Round1_item24	8.666666667	0.887625365	Round2_item24	8.636363636	0.924416278
USER	Round1_item24	7.551724138	1.325200979	Round2_item24	7.2	1.105012503
MIDWIFES	Round1_item25	8	1.143958905	Round2_item25	8	1.175735064
RESEARCHER	Round1_item25	8.583333333	0.900336637	Round2_item25	8.636363636	0.924416278
USER	Round1_item25	7.689655172	1.416823617	Round2_item25	7.380952381	1.203170415
MIDWIFES	Round1_item26	7.512195122	1.316972796	Round2_item26	**7.637681159**	1.339128497
RESEARCHER	Round1_item26	8.166666667	1.337115847	Round2_item26	**8.181818182**	1.401298099
USER	Round1_item26	7.066666667	1.507071454	Round2_item26	**6.590909091**	1.402687587
MIDWIFES	Round1_item27	8.353658537	0.907743794	Round2_item27	8.391304348	0.878122853
RESEARCHER	Round1_item27	8.666666667	0.887625365	Round2_item27	8.727272727	0.904534034
USER	Round1_item27	8.032258065	1.353609273	Round2_item27	7.590909091	1.501081861
MIDWIFES	Round1_item28	8.512195122	0.835092993	Round2_item28	8.565217391	0.776081046
RESEARCHER	Round1_item28	8.666666667	0.887625365	Round2_item28	8.727272727	0.904534034
USER	Round1_item28	8.166666667	1.205828756	Round2_item28	7.80952381	1.249761882
MIDWIFES	Round1_item29	7.963414634	1.023732242	Round2_item29	8	1.014599312
RESEARCHER	Round1_item29	8.333333333	1.497472618	Round2_item29	8.363636364	1.566698904
USER	Round1_item29	7.466666667	1.431982791	Round2_item29	7.090909091	1.411149174
MIDWIFES	Round1_item31	7.5	1.372121004	Round2_item31	7.507246377	1.461497906
RESEARCHER	Round1_item31	8.25	1.422226168	Round2_item31	8.272727273	1.489356176
USER	Round1_item31	7.5	1.414213562	Round2_item31	7.166666667	1.339446769
MIDWIFES	Round1_item32	7.170731707	1.27468106	Round2_item32	**7.188405797**	1.331467151
RESEARCHER	Round1_item32	8.083333333	1.56427929	Round2_item32	**8.090909091**	1.640399065
USER	Round1_item32	7	1.732050808	Round2_item32	**6.9**	1.293709477
MIDWIFES	Round1_item33	7.585365854	1.360385017	Round2_item33	7.608695652	1.447283371
RESEARCHER	Round1_item33	8.25	1.422226168	Round2_item33	8.272727273	1.489356176
USER	Round1_item33	7.444444444	1.423250163	Round2_item33	7.2	1.361114095
MIDWIFES	Round1_item34	7.414634146	1.304798547	Round2_item34	7.420289855	1.365604769
RESEARCHER	Round1_item34	8.25	1.422226168	Round2_item34	8.272727273	1.489356176
USER	Round1_item34	7	1.64924225	Round2_item34	7.105263158	1.149624907
MIDWIFES	Round1_item36	**7.756097561**	1.262576003	Round2_item36	**7.782608696**	1.293303627
RESEARCHER	Round1_item36	**8.25**	1.422226168	Round2_item36	**8.454545455**	1.293339581
USER	Round1_item36	**6.866666667**	1.795268366	Round2_item36	**6.5**	1.535298947
MIDWIFES	Round1_item37	**7**	1.735611039	Round2_item37	**7.130434783**	1.542658548
RESEARCHER	Round1_item37	**8.25**	1.356801051	Round2_item37	**8.272727273**	1.420627262
USER	Round1_item37	**6.448275862**	1.956421787	Round2_item37	**5.909090909**	1.659291039
MIDWIFES	Round1_item38	7.719512195	1.199530672	Round2_item38	**7.710144928**	1.237899659
RESEARCHER	Round1_item38	8.5	0.797724035	Round2_item38	**8.454545455**	0.820199532
USER	Round1_item38	7.066666667	1.760355241	Round2_item38	**6.772727273**	1.688386612
MIDWIFES	Round1_item39	8.317073171	0.928001288	Round2_item39	8.362318841	0.923086486

RESEARCHER	Round1_item39	8.666666667	1.154700538	Round2_item39	8.636363636	1.206045378
USER	Round1_item39	7.903225806	1.513381175	Round2_item39	7.5	1.503963019
MIDWIFES	Round1_item40	7.719512195	1.080402191	Round2_item40	7.724637681	1.123074273
RESEARCHER	Round1_item40	8.583333333	0.792961461	Round2_item40	8.545454545	0.820199532
USER	Round1_item40	7.766666667	1.040004421	Round2_item40	7.590909091	0.959121171
MIDWIFES	Round1_item41	8.06097561	1.034557948	Round2_item41	8.101449275	1.045226228
RESEARCHER	Round1_item41	8.75	0.621581561	Round2_item41	8.727272727	0.646669791
USER	Round1_item41	7.533333333	1.382983615	Round2_item41	7.272727273	1.31590339
MIDWIFES	Round1_item42	8.207317073	0.978002561	Round2_item42	8.260869565	0.994871761
RESEARCHER	Round1_item42	8.75	0.621581561	Round2_item42	8.727272727	0.646669791
USER	Round1_item42	8.066666667	1.2298958	Round2_item42	7.909090909	1.269011273
MIDWIFES	Round1_item44	7.426829268	1.474310785	Round2_item44	**7.376811594**	1.563381039
RESEARCHER	Round1_item44	8.333333333	1.497472618	Round2_item44	**8.272727273**	1.555050423
USER	Round1_item44	7.153846154	1.541228281	Round2_item44	**6.894736842**	1.523692038
MIDWIFES	Round1_item45	**7.280487805**	1.269532725	Round2_item45	**7.260869565**	1.324566232
RESEARCHER	Round1_item45	**8.083333333**	1.621353718	Round2_item45	**8**	1.673320053
USER	Round1_item45	**6.703703704**	1.539600718	Round2_item45	**6.55**	1.145931017
MIDWIFES	Round1_item46	7.56097561	1.458034877	Round2_item46	**7.536231884**	1.549000723
RESEARCHER	Round1_item46	8.333333333	1.497472618	Round2_item46	**8.272727273**	1.555050423
USER	Round1_item46	7.230769231	1.58259767	Round2_item46	**6.894736842**	1.523692038
MIDWIFES	Round1_item47	**7.548780488**	1.218706449	Round2_item47	**7.52173913**	1.255677643
RESEARCHER	Round1_item47	**8.333333333**	1.497472618	Round2_item47	**8.272727273**	1.555050423
USER	Round1_item47	**6.925925926**	1.491667264	Round2_item47	**6.75**	1.251315098
MIDWIFES	Round1_item48	7.963414634	1.328185637	Round2_item48	8.072463768	0.98993227
RESEARCHER	Round1_item48	8.833333333	0.577350269	Round2_item48	8.818181818	0.603022689
USER	Round1_item48	7.966666667	1.376736104	Round2_item48	7.857142857	1.458962449
MIDWIFES	Round1_item49	8.134146341	1.003231755	Round2_item49	8.130434783	0.998720409
RESEARCHER	Round1_item49	8.833333333	0.577350269	Round2_item49	8.818181818	0.603022689
USER	Round1_item49	7.866666667	1.357821108	Round2_item49	7.761904762	1.220850601
MIDWIFES	Round1_item51	**6.170731707**	1.600963275	Round2_item51	**6.333333333**	1.379115943
RESEARCHER	Round1_item51	**7.666666667**	1.556997888	Round2_item51	**7.545454545**	1.572490786
USER	Round1_item51	**7**	1.845916414	Round2_item51	**6.6**	1.667017507
MIDWIFES	Round1_item56	8.475609756	0.849482192	Round2_item56	8.52173913	0.833290706
RESEARCHER	Round1_item56	8.25	1.484771179	Round2_item56	8.181818182	1.53741223
USER	Round1_item56	7.321428571	1.415615857	Round2_item56	7.1	1.447320573
MIDWIFES	Round1_item57	**6.024390244**	2.926994876	Round2_item57	**6.31884058**	2.757304489
RESEARCHER	Round1_item57	**7.666666667**	1.61432977	Round2_item57	**7.636363636**	1.689540013
USER	Round1_item57	**6.2**	1.823819012	Round2_item57	**5.733333333**	1.751190072
MIDWIFES	Round1_item59	7.987804878	1.02432034	Round2_item59	8.014492754	1.064020588
RESEARCHER	Round1_item59	8.25	1.712255291	Round2_item59	8.272727273	1.793929156
USER	Round1_item59	7.533333333	1.407696415	Round2_item59	7.285714286	1.007117528
MIDWIFES	Round1_item60	**7.256097561**	1.554054638	Round2_item60	**7.304347826**	1.611578946
RESEARCHER	Round1_item60	**7.5**	2.110579412	Round2_item60	**7.363636364**	2.15743956
USER	Round1_item60	**6.260869565**	2.049775848	Round2_item60	**5.823529412**	1.845104811

MIDWIFES	Round1_item61	**6.548780488**	1.931879652	Round2_item61	**6.710144928**	1.831918388
RESEARCHER	Round1_item61	**7.583333333**	1.621353718	Round2_item61	**7.454545455**	1.634847783
USER	Round1_item61	**6.466666667**	1.696514344	Round2_item61	**6.19047619**	1.503963019
MIDWIFES	Round1_item62	**7.243902439**	1.902311626	Round2_item62	**7.405797101**	1.784894465
RESEARCHER	Round1_item62	**8.25**	1.422226168	Round2_item62	**8.181818182**	1.470930441
USER	Round1_item62	**6.947368421**	1.649029486	Round2_item62	**6.846153846**	1.28102523
MIDWIFES	Round1_item68	8.036585366	1.082351372	Round2_item68	8.028985507	1.084259228
RESEARCHER	Round1_item68	8.583333333	0.900336637	Round2_item68	8.545454545	0.934198733
USER	Round1_item68	7.966666667	1.32569652	Round2_item68	7.428571429	1.535298947
MIDWIFES	Round1_item69	8.317073171	1.255880477	Round2_item69	8.31884058	1.322795098
RESEARCHER	Round1_item69	8.583333333	0.900336637	Round2_item69	8.545454545	0.934198733
USER	Round1_item69	8.033333333	1.32569652	Round2_item69	7.619047619	1.160869953
MIDWIFES	Round1_item77	8.182926829	1.388915989	Round2_item77	8.260869565	1.441528742
RESEARCHER	Round1_item77	8.666666667	0.651338947	Round2_item77	8.636363636	0.674199862
USER	Round1_item77	7.655172414	1.674938423	Round2_item77	7.285714286	1.82051798
MIDWIFES	Round1_item79	8.231707317	0.959665329	Round2_item79	8.260869565	0.9799786
RESEARCHER	Round1_item79	8.583333333	0.99620492	Round2_item79	8.545454545	1.035725481
USER	Round1_item79	7.9	1.493664783	Round2_item79	7.636363636	1.528941574
MIDWIFES	Round1_item80	7.707317073	1.888809181	Round2_item80	7.971014493	1.484934287
RESEARCHER	Round1_item80	8.833333333	0.389249472	Round2_item80	8.818181818	0.404519917
USER	Round1_item80	7.5	1.819209666	Round2_item80	7.470588235	1.662740474
MIDWIFES	Round1_item81	7.670731707	1.482864079	Round2_item81	7.724637681	1.580060107
RESEARCHER	Round1_item81	8.583333333	0.792961461	Round2_item81	8.545454545	0.820199532
USER	Round1_item81	7.566666667	1.612095089	Round2_item81	7.227272727	1.477830398
MIDWIFES	Round1_item82	8.170731707	1.063477618	Round2_item82	8.289855072	0.986697541
RESEARCHER	Round1_item82	8.583333333	0.668557923	Round2_item82	8.545454545	0.687551651
USER	Round1_item82	7.75862069	1.661650909	Round2_item82	7.571428571	1.567527626
MIDWIFES	Round1_item83	**7.524390244**	1.806884022	Round2_item83	**7.579710145**	1.709883012
RESEARCHER	Round1_item83	**8**	1.348399725	Round2_item83	**8.090909091**	1.375103302
USER	Round1_item83	**6.458333333**	2.146365313	Round2_item83	**6.294117647**	2.143732314
MIDWIFES	Round1_item88	**5.646341463**	2.353730694	Round2_item88	**5.768115942**	2.377250807
RESEARCHER	Round1_item88	**7.25**	2.00567377	Round2_item88	**7.363636364**	2.062654953
USER	Round1_item88	**7.041666667**	1.517411029	Round2_item88	**6.5625**	1.209338662
MIDWIFES	Round1_item90	7.341463415	1.664587683	Round2_item90	7.31884058	1.761456229
RESEARCHER	Round1_item90	8.5	1.167748416	Round2_item90	8.454545455	1.213559752
USER	Round1_item90	7.428571429	1.47644647	Round2_item90	7.047619048	1.283596139
MIDWIFES	Round1_item96	**6.512195122**	2.485731641	Round2_item96	**6.724637681**	2.484564454
RESEARCHER	Round1_item96	**7.916666667**	1.505042031	Round2_item96	**7.818181818**	1.53741223
USER	Round1_item96	**6.740740741**	2.067934544	Round2_item96	**6**	2.029198625
MIDWIFES	Round1_item97	7.87804878	1.058653278	Round2_item97	7.913043478	1.081109594
RESEARCHER	Round1_item97	8.583333333	0.900336637	Round2_item97	8.545454545	0.934198733
USER	Round1_item97	7.8	1.186126701	Round2_item97	7.476190476	1.123345344
MIDWIFES	Round1_item98	8.56097561	1.155743158	Round2_item98	8.579710145	1.217589194
RESEARCHER	Round1_item98	8.166666667	1.642245322	Round2_item98	8.090909091	1.700267359

USER	Round1_item98	8.24137931	1.272095358	Round2_item98	7.85	1.386969434
MIDWIFES	Round1_item99	8.475609756	1.239530076	Round2_item99	8.52173913	1.278886184
RESEARCHER	Round1_item99	8	1.651445648	Round2_item99	7.909090909	1.700267359
USER	Round1_item99	7.964285714	1.477789712	Round2_item99	7.578947368	1.538967528
MIDWIFES	Round1_item100	**8.329268293**	1.25761756	Round2_item100	**8.420289855**	1.288019422
RESEARCHER	Round1_item100	**7.833333333**	2.124888589	Round2_item100	**7.727272727**	2.195035721
USER	Round1_item100	**6.8125**	1.905037182	Round2_item100	**6.384615385**	1.804552647
MIDWIFES	Round1_item102	7.634146341	1.51137412	Round2_item102	7.782608696	1.270358509
RESEARCHER	Round1_item102	8.416666667	0.99620492	Round2_item102	8.454545455	1.035725481
USER	Round1_item102	7.225806452	1.927168517	Round2_item102	7.5	1.471960144
MIDWIFES	Round1_item103	**6.975609756**	2.293176188	Round2_item103	**7**	2.307277489
RESEARCHER	Round1_item103	**8.166666667**	1.193416283	Round2_item103	**8.181818182**	1.250454463
USER	Round1_item103	**6.695652174**	2.054590926	Round2_item103	**6.6875**	1.400892573
MIDWIFES	Round1_item105	8.268292683	1.247701892	Round2_item105	8.304347826	1.275381694
RESEARCHER	Round1_item105	8.583333333	0.900336637	Round2_item105	8.545454545	0.934198733
USER	Round1_item105	7.285714286	1.921749644	Round2_item105	7.272727273	1.548634366
MIDWIFES	Round1_item106	8.292682927	0.909318085	Round2_item106	8.304347826	0.912403868
RESEARCHER	Round1_item106	8.083333333	1.378954369	Round2_item106	8	1.414213562
USER	Round1_item106	7.5	1.749285568	Round2_item106	7.631578947	1.211542924
MIDWIFES	Round1_item108	7.731707317	1.042892233	Round2_item108	7.768115942	1.031060669
RESEARCHER	Round1_item108	8.25	1.422226168	Round2_item108	8.181818182	1.470930441
USER	Round1_item108	7.222222222	1.739436985	Round2_item108	7.263157895	1.62761261
MIDWIFES	Round1_item109	8.182926829	1.101380153	Round2_item109	8.246376812	1.020464097
RESEARCHER	Round1_item109	8.666666667	0.887625365	Round2_item109	8.636363636	0.924416278
USER	Round1_item109	7.724137931	1.436676515	Round2_item109	7.65	1.268027893
MIDWIFES	Round1_item125	8.048780488	0.928325707	Round2_item125	8.086956522	0.935243448
RESEARCHER	Round1_item125	8.25	1.055289706	Round2_item125	8.181818182	1.07871978
USER	Round1_item125	7.678571429	1.492042384	Round2_item125	7.2	1.542383664
MIDWIFES	Round1_item127	8.097560976	0.988948264	Round2_item127	8.144927536	0.98928617
RESEARCHER	Round1_item127	8.416666667	0.99620492	Round2_item127	8.454545455	1.035725481
USER	Round1_item127	7.866666667	1.332183412	Round2_item127	7.666666667	1.316561177
MIDWIFES	Round1_item129	7.804878049	1.115978499	Round2_item129	7.826086957	1.149891577
RESEARCHER	Round1_item129	8.25	0.965307299	Round2_item129	8.181818182	0.981649817
USER	Round1_item129	7.461538462	1.859693937	Round2_item129	7.142857143	1.878449208
MIDWIFES	Round1_item130	7.670731707	1.361325408	Round2_item130	**7.797101449**	1.356679664
RESEARCHER	Round1_item130	8.166666667	1.029857301	Round2_item130	**8.090909091**	1.044465936
USER	Round1_item130	7.464285714	1.794686455	Round2_item130	**6.95**	1.848897253
MIDWIFES	Round1_item132	7.524390244	1.316801304	Round2_item132	7.579710145	1.37633141
RESEARCHER	Round1_item132	8.166666667	1.193416283	Round2_item132	8.090909091	1.221027883
USER	Round1_item132	7.290322581	1.487583015	Round2_item132	7.227272727	1.509708839
MIDWIFES	Round1_item133	8.134146341	1.312449365	Round2_item133	8.246376812	1.354793202
RESEARCHER	Round1_item133	8.583333333	0.792961461	Round2_item133	8.545454545	0.820199532
USER	Round1_item133	7.64516129	1.473055489	Round2_item133	7.409090909	1.333062743
MIDWIFES	Round1_item134	8.048780488	1.017166714	Round2_item134	8.202898551	0.963750489

RESEARCHER	Round1_item134	8.416666667	1.083624669	Round2_item134	8.363636364	1.120064933
USER	Round1_item134	7.709677419	1.321452443	Round2_item134	7.409090909	1.296849329
MIDWIFES	Round1_item135	7.804878049	1.435347632	Round2_item135	7.869565217	1.51378998
RESEARCHER	Round1_item135	8.416666667	1.083624669	Round2_item135	8.363636364	1.120064933
USER	Round1_item135	7.225806452	1.407353957	Round2_item135	7.227272727	1.269863817
MIDWIFES	Round1_item136	**7.646341463**	1.605330251	Round2_item136	**7.608695652**	1.699631373
RESEARCHER	Round1_item136	**7.5**	1.732050808	Round2_item136	**7.363636364**	1.747725795
USER	Round1_item136	**6.652173913**	1.897574903	Round2_item136	**6.176470588**	1.810914621
MIDWIFES	Round1_item141	8.268292683	0.943447585	Round2_item141	8.333333333	0.934103324
RESEARCHER	Round1_item141	8.666666667	0.778498944	Round2_item141	8.636363636	0.809039835
USER	Round1_item141	7.068965517	1.907336109	Round2_item141	7.142857143	1.711306936
MIDWIFES	Round1_item149	7.780487805	1.247460534	Round2_item149	**7.797101449**	1.278552837
RESEARCHER	Round1_item149	7.75	1.712255291	Round2_item149	**7.636363636**	1.747725795
USER	Round1_item149	7.333333333	1.340560125	Round2_item149	**6.764705882**	1.200490096
MIDWIFES	Round1_item154	7.256097561	2.035554195	Round2_item154	7.260869565	2.146489432
RESEARCHER	Round1_item154	8.25	1.356801051	Round2_item154	8.272727273	1.420627262
USER	Round1_item154	8	1.183215957	Round2_item154	7.636363636	1.292669978
MIDWIFES	Round1_item156	7.170731707	2.243260843	Round2_item156	7.130434783	2.418756629
RESEARCHER	Round1_item156	7.916666667	1.164500153	Round2_item156	8.090909091	1.044465936
USER	Round1_item156	8.064516129	1.289327573	Round2_item156	7.454545455	1.335496081
MIDWIFES	Round1_item157	7.243902439	2.111467664	Round2_item157	7.275362319	2.092592383
RESEARCHER	Round1_item157	7.833333333	1.337115847	Round2_item157	8	1.264911064
USER	Round1_item157	8.064516129	1.093480189	Round2_item157	7.863636364	0.990211836
MIDWIFES	Round1_item158	**6.780487805**	2.298815531	Round2_item158	**6.826086957**	2.344935229
RESEARCHER	Round1_item158	**8**	1.279204298	Round2_item158	**8.090909091**	1.300349603
USER	Round1_item158	**7.866666667**	1.224275531	Round2_item158	**7.636363636**	1.328997278
MIDWIFES	Round1_item159	**7.268292683**	1.778116499	Round2_item159	**7.304347826**	1.904363008
RESEARCHER	Round1_item159	**8**	1.477097892	Round2_item159	**7.909090909**	1.513574937
USER	Round1_item159	**6.967741935**	1.663006734	Round2_item159	**6.5**	1.471960144
MIDWIFES	Round1_item160	6.93902439	1.694927516	Round2_item160	**7.057971014**	1.625930123
RESEARCHER	Round1_item160	6.25	2.927145678	Round2_item160	**6.454545455**	2.978712351
USER	Round1_item160	7.6	1.248447312	Round2_item160	**7.19047619**	1.209092537
MIDWIFES	Round1_item162	7.463414634	1.847220949	Round2_item162	7.434782609	1.966470867
RESEARCHER	Round1_item162	8.416666667	0.99620492	Round2_item162	8.363636364	1.026910636
USER	Round1_item162	7.483870968	1.363108402	Round2_item162	7.090909091	1.341963413
MIDWIFES	Round1_item164	7.768292683	1.597055783	Round2_item164	7.768115942	1.707637924
RESEARCHER	Round1_item164	8.833333333	0.389249472	Round2_item164	8.818181818	0.404519917
USER	Round1_item164	7.862068966	1.125171032	Round2_item164	7.7	1.080935268

Legend: sd= standard deviation. In bold the items with a significant mean differences.

Python syntax ANOVA mixed

```
import pandas as pd

import statsmodels.api as sm

from statsmodels.formula.api import ols


# Carica il file SPSS

file_path = "prova.sav"

df = pd.read_spss(file_path)

print(df.columns)


# Lista per salvare i risultati

results = []


# Loop su tutti i 165 item

for i in range(1, 166):

    item = f"Round1_item{i}"

    item_time2 = f"Round2_item{i}"


    # Riorganizza i dati per avere una colonna unica per i valori dell'item nei due tempi

    item_df = df[['Category', item, item_time2]].melt(id_vars=['Category'], var_name='time',
value_name='value').dropna()


    # Adatta la colonna 'time' per riconoscere i due tempi di misurazione

    item_df['time'] = item_df['time'].apply(lambda x: 'Time1' if 'Round1' in x else 'Time2')


    # Crea il modello ANOVA
```

```
    model = ols('value ~ C(Category) * C(time)', data=item_df).fit()
    anova_table = sm.stats.anova_lm(model, typ=2)
    print(anova_table)  # Visualizza la tabella ANOVA per verificare i nomi delle colonne

    # Salva i risultati
    results.append({
        "Item": item,
        "Category p-value": anova_table["PR(>F)"]["C(Category)"],
        "Time p-value": anova_table["PR(>F)"]["C(time)"],
        "Interaction p-value": anova_table["PR(>F)"]["C(Category):C(time)"]
    })

# Converte i risultati in DataFrame e salva in Excel
results_df = pd.DataFrame(results)
output_path = "anova_results_prova.xlsx"
results_df.to_excel(output_path, index=False)
print(f"Risultati salvati in {output_path}")
import pandas as pd

# Carica il file Excel
df = pd.read_excel("anova_results_prova.xlsx")

# Controlla quanti p-value sono significativi (< 0.05) per ogni colonna
significativi_category = df[df["Category p-value"] < 0.05]
significativi_time = df[df["Time p-value"] < 0.05]
significativi_interaction = df[df["Interaction p-value"] < 0.05]
```

```
# Conta il numero di p-value significativi per ogni colonna
count_category = significativi_category.shape[0]
count_time = significativi_time.shape[0]
count_interaction = significativi_interaction.shape[0]

# Stampa i risultati
print(f"Numero di item con p-value significativo per Category: {count_category}")
print(f"Numero di item con p-value significativo per Time: {count_time}")
print(f"Numero di item con p-value significativo per Interaction: {count_interaction}")

# Opzionale: stampa gli item significativi per ogni colonna
print("\nItem significativi per Category:")
print(significativi_category["Item"].tolist())

print("\nItem significativi per Time:")
print(significativi_time["Item"].tolist())

print("\nItem significativi per Interaction:")
print(significativi_interaction["Item"].tolist())

import pandas as pd

# Carica il file SPSS
file_path = 'C:/Users/arian/OneDrive - Università degli Studi di Milano/prova/prova.sav'
df = pd.read_spss(file_path)

# Lista degli item senza prefisso
```

```
item_list = [
    'item2', 'item3', 'item5', 'item6', 'item7', 'item8', 'item9', 'item10', 'item14',
    'item16', 'item17', 'item18', 'item20', 'item21', 'item22', 'item24', 'item25',
    'item26', 'item27', 'item28', 'item29', 'item31', 'item32', 'item33', 'item34',
    'item36', 'item37', 'item38', 'item39', 'item40', 'item41', 'item42', 'item44',
    'item45', 'item46', 'item47', 'item48', 'item49', 'item51', 'item56', 'item57',
    'item59', 'item60', 'item61', 'item62', 'item68', 'item69', 'item77', 'item79',
    'item80', 'item81', 'item82', 'item83', 'item88', 'item90', 'item96', 'item97',
    'item98', 'item99', 'item100', 'item102', 'item103', 'item105', 'item106', 'item108',
    'item109', 'item125', 'item127', 'item129', 'item130', 'item132', 'item133',
    'item134', 'item135', 'item136', 'item141', 'item149', 'item154', 'item156',
    'item157', 'item158', 'item159', 'item160', 'item162', 'item164'
]

# Costruisci le liste di item per Round1 e Round2
round1_items = [f'Round1_{item}' for item in item_list]
round2_items = [f'Round2_{item}' for item in item_list]

# Lista per salvare i risultati
results = []

# Ciclo su ogni item per confrontare Round1 e Round2 per Category
for r1_item, r2_item in zip(round1_items, round2_items):
    # Calcola statistiche descrittive per Round1
    round1_stats = df.groupby('Category')[r1_item].agg(['mean', 'std']).reset_index()
    round1_stats['Round'] = 'Round1'
    round1_stats['Item'] = r1_item
```

```
    # Calcola statistiche descrittive per Round2
    round2_stats = df.groupby('Category')[r2_item].agg(['mean', 'std']).reset_index()
    round2_stats['Round'] = 'Round2'
    round2_stats['Item'] = r2_item

    # Combina i risultati di Round1 e Round2 in colonne affiancate
    combined_stats = pd.merge(
        round1_stats,
        round2_stats,
        on="Category",
        suffixes=('_Round1', '_Round2')
    )

    # Aggiungi ai risultati complessivi
    results.append(combined_stats)

# Combina tutti i risultati in un unico DataFrame
final_results = pd.concat(results, ignore_index=True)

# Salva i risultati descrittivi in un file Excel
output_path = 'output_descrittivi_confronto_rounds_riformattato.xlsx'  # Nome del file di output
final_results.to_excel(output_path, index=False)

print(f"Risultati salvati in: {output_path}")
```
