# Supplementary material for: Development and Validation of the Midwifery Interventions Classification for a Salutogenic Approach to Maternity Care: A Delphi Study
Source: Healthcare (Basel). 2024 Nov 8;12(22):2228. doi: 10.3390/healthcare12222228 (PMC11594468; doi:10.3390/healthcare12222228)
Supplement: Supplementary file 1 [file healthcare-12-02228-s001.zip › Table S3.pdf]

Table S3: Suggested additional midwifery interventions and proposed modifications from Round 1 Delphi survey to consider for presentation in Round 2

Suggestions and comments by Delphi participants in Round 1 (transcription)	Proposed modification by authors
**Midwives Panel**	
"Communication adapted to the patient"	Concept already present in the MIC through different midwifery interventions: *Personalized care*, *Counselling,* and *Promotion of health literacy.*
"Sex education and promotion of informed pregnancy planning"	Two midwifery interventions modified both in titles and definitions: *Contraception and family planning counselling* and *Sexual and reproductive health counselling*.
"Consideration of the family context"	New midwifery intervention added: *Consideration of the family context*.
**Healthcare Researchers Panel**	
"I would use the term *latent phase* in the early labour in place of *prodromal period*"	One midwifery intervention modified in the title: *Early labour care*.
"Policies and practical support for the families and standardization and promotion of paternity policies"	Concept already present in the MIC through one midwifery interventions: *Monitoring of health policy.*
**Maternity Service Users Panel**	
"Prevention, first and foremost, is through education for healthy nutrition and lifestyles of future mothers and fathers. Economic resources must be adequate for ambitious health policies. We need more investments in the technical and emotional education of healthcare professionals. It is necessary to consolidate empathy and human respect between patients and healthcare."	Concepts already present in the MIC through different midwifery interventions: *Counselling, Healthy behaviour counselling, Health education, Monitoring of health policy*. New midwifery intervention added: *Humanization of care*.
"I think it is essential for a more thorough education of healthcare professionals on the *human* aspect of the patient, which has been completely missed in my birth experience."	New midwifery intervention added: *Humanization of care*.
