# Supplementary material for: Development and Validation of the Midwifery Interventions Classification for a Salutogenic Approach to Maternity Care: A Delphi Study
Source: Healthcare (Basel). 2024 Nov 8;12(22):2228. doi: 10.3390/healthcare12222228 (PMC11594468; doi:10.3390/healthcare12222228)
Supplement: Supplementary file 1 [file healthcare-12-02228-s001.zip › Table S4.pdf]

Table S4. Summary of Delphi rounds results

Midwifery interventions	ROUND 1												Round 1 results	ROUND 2												Round 2 results	FINAL DECISION
	Midwives			Healthcare Researchers			Maternity Service Users			Overall				Midwives			Healthcare Researchers			Maternity Service Users			Overall				
	Not important (%)	Important but not critical (%)	Critical (%)	Not important (%)	Important but not critical (%)	Critical (%)	Not important (%)	Important but not critical (%)	Critical (%)	Not important (%)	Important but not critical (%)	Critical (%)		Not important (%)	Important but not critical (%)	Critical (%)	Not important (%)	Important but not critical (%)	Critical (%)	Not important (%)	Important but not critical (%)	Critical (%)	Not important (%)	Important but not critical (%)	Critical (%)		
Active listening	0	1	98	0	0	100	0	0	97	0	1	98	Consensus for inclusion	0	1	99	0	0	100	0	0	100	0	1	99	Consensus for inclusion	**Included**
Administration of analgesia	1	35	61	0	17	83	0	23	61	1	30	63	No consensus	0	26	74	0	27	73	0	41	59	0	29	71	Consensus for inclusion	**Included**
Administration of medication	0	28	70	8	8	83	0	19	65	1	24	70	Consensus for inclusion	0	17	83	0	9	91	0	36	64	0	20	80	Consensus for inclusion	**Included**
Advocate for respectful perinatal care	0	5	94	0	8	92	0	10	90	0	6	93	Consensus for inclusion	0	0	99	0	9	91	0	5	91	0	2	96	Consensus for inclusion	**Included**
Allergy management	2	20	73	0	8	75	3	13	65	2	17	71	Consensus for inclusion	0	11	89	0	9	91	0	23	77	0	14	86	Consensus for inclusion	**Included**
Antenatal care	0	0	99	0	0	100	0	23	74	0	6	93	Consensus for inclusion	0	0	100	0	0	100	0	18	82	0	4	96	Consensus for inclusion	**Included**
Antenatal education*	0	13	85	0	8	92	0	13	84	0	13	86	Consensus for inclusion	0	7	93	0	0	100	0	9	91	0	7	93	Consensus for inclusion	**Included**
Assessment of new equipment	1	21	77	0	42	58	3	16	61	2	22	71	Consensus for inclusion	0	11	87	0	18	82	0	27	68	0	16	83	Consensus for inclusion	**Included**

Assessment of uterine contractions	0	1	96	8	8	83	3	16	32	2	6	79	Consensus for inclusion	0	1	99	0	0	100	0	14	86	0	4	96	Consensus for inclusion	**Included**
Birth care	0	0	98	0	0	100	0	0	97	0	0	98	Consensus for inclusion	0	0	100	0	0	100	0	0	100	0	0	100	Consensus for inclusion	**Included**
Body temperature regulation	0	10	85	0	17	83	6	35	35	2	17	73	Consensus for inclusion	0	11	89	0	9	91	0	45	55	0	18	82	Consensus for inclusion	**Included**
Boosting self-confidence	0	24	73	0	17	83	0	29	71	0	25	74	Consensus for inclusion	0	13	87	0	18	82	5	41	55	1	19	80	Consensus for inclusion	**Included**
Bottle feeding	16	54	28	8	42	50	3	26	58	12	46	38	No consensus	7	76	17	9	55	36	0	36	64	6	65	29	No consensus	**Excluded**
Breastfeeding counselling*	0	2	96	0	8	92	0	6	90	0	4	94	Consensus for inclusion	0	0	100	0	9	91	0	5	95	0	2	98	Consensus for inclusion	**Included**
Budget management	1	12	80	0	17	83	0	13	87	1	13	82	Consensus for inclusion	0	16	83	0	9	91	0	27	68	0	17	81	Consensus for inclusion	**Included**
Capillary blood sampling	1	35	52	8	8	83	0	23	39	2	30	52	No consensus	0	43	57	9	27	64	9	55	36	3	44	53	No consensus	**Excluded**
Care at home	0	18	80	0	8	92	3	26	71	1	19	79	Consensus for inclusion	0	11	87	0	9	91	0	32	64	0	16	83	Consensus for inclusion	**Included**
Care planning	0	7	93	0	8	92	0	23	77	0	11	89	Consensus for inclusion	0	3	96	0	0	100	0	27	68	0	8	90	Consensus for inclusion	**Included**
Caregiver engagement	0	16	84	0	8	83	6	19	68	2	16	80	Consensus for inclusion	0	9	90	0	18	82	0	18	77	0	12	86	Consensus for inclusion	**Included**
Caregiver support	0	35	60	0	8	83	3	19	65	1	29	63	No consensus	0	44	56	0	27	73	0	36	64	0	41	59	No consensus	**Excluded**
Case management	0	18	74	0	8	92	3	35	35	1	22	66	Consensus for inclusion	0	20	79	0	18	82	0	45	50	0	25	73	Consensus for inclusion	**Included**
Clinical record-keeping	0	12	87	0	8	92	0	16	74	0	13	84	Consensus for inclusion	0	7	91	0	0	100	0	9	86	0	7	91	Consensus for inclusion	**Included**
Communication of healthcare information	0	17	82	0	8	92	0	29	68	0	19	79	Consensus for inclusion	0	11	87	0	9	91	0	9	86	0	11	87	Consensus for inclusion	**Included**

Consideration of the family context	Midwifery intervention added after the Round 1													0	20	80	0	9	91	0	41	59	0	23	77	Consensus for inclusion	**Included**
Contraception and family planning counselling*	1	35	61	0	17	83	10	35	48	3	34	60	No consensus	0	14	86	0	9	91	5	64	32	1	24	75	Consensus for inclusion	**Included**
Coping enhancement	0	23	72	0	8	92	0	32	29	0	24	63	Consensus for inclusion	0	19	81	0	18	82	0	59	41	0	27	73	Consensus for inclusion	**Included**
Cord clamping	4	15	79	0	17	83	3	10	65	3	14	76	Consensus for inclusion	1	13	86	0	9	91	0	23	77	1	15	84	Consensus for inclusion	**Included**
Counselling	0	4	95	0	8	92	0	19	74	0	8	90	Consensus for inclusion	0	0	100	0	9	91	0	32	68	0	8	92	Consensus for inclusion	**Included**
Counselling on newborn safety	0	2	96	0	8	92	0	13	87	0	6	94	Consensus for inclusion	0	0	100	0	0	100	0	5	95	0	1	99	Consensus for inclusion	**Included**
Counselling pregnant people on respectful maternity care	0	15	84	0	8	92	0	16	77	0	14	83	Consensus for inclusion	0	4	96	0	0	100	0	23	77	0	8	92	Consensus for inclusion	**Included**
Counselling the birthing person on their own bowel movements	0	29	70	0	17	83	3	32	52	1	29	66	No consensus	0	33	67	0	9	91	0	55	45	0	35	65	Consensus for inclusion	**Included**
Counselling the birthing person on their own urinary elimination	1	21	77	0	8	92	3	26	55	2	21	73	Consensus for inclusion	0	14	86	0	9	91	0	41	59	0	19	81	Consensus for inclusion	**Included**
Cultural mediation	0	18	82	0	8	92	3	29	58	1	20	77	Consensus for inclusion	0	9	90	0	18	82	0	9	86	0	10	88	Consensus for inclusion	**Included**
Cup feeding	12	41	43	8	25	67	29	19	23	16	34	40	Consensus for exclusion	4	67	29	18	55	27	32	55	14	12	63	25	No consensus	**Excluded**
Data collection for research purposes	0	12	84	0	8	92	0	26	74	0	15	82	Consensus for inclusion	0	13	86	0	0	100	0	23	73	0	14	84	Consensus for inclusion	**Included**
Decision-making support	0	17	79	0	8	92	0	26	65	0	18	77	Consensus for inclusion	0	17	83	0	18	82	5	18	77	1	17	82	Consensus for inclusion	**Included**
Development of health programmes	0	9	84	8	0	92	0	26	68	1	12	81	Consensus for inclusion	0	17	81	0	9	91	0	27	68	0	18	80	Consensus for inclusion	**Included**

Diagnostic test prescription	0	24	74	0	25	75	3	19	68	1	23	73	Consensus for inclusion	0	17	81	0	0	100	0	23	73	0	17	82	Consensus for inclusion	**Included**
Discharge planning	0	13	85	0	8	92	0	32	68	0	18	82	Consensus for inclusion	0	7	91	0	9	91	0	18	77	0	10	88	Consensus for inclusion	**Included**
Early labour care*	1	12	83	0	25	75	10	16	71	3	14	79	Consensus for inclusion	0	11	89	0	18	82	0	36	64	0	17	83	Consensus for inclusion	**Included**
Early postnatal observation	0	1	96	0	8	92	0	13	84	0	5	93	Consensus for inclusion	0	1	99	0	9	91	0	0	100	0	2	98	Consensus for inclusion	**Included**
Emergency trolley check	0	6	94	8	17	75	0	13	65	1	9	85	Consensus for inclusion	0	6	93	0	0	100	0	14	82	0	7	91	Consensus for inclusion	**Included**
Emotional support	0	9	89	0	8	92	0	13	84	0	10	88	Consensus for inclusion	0	6	94	0	0	100	0	9	91	0	6	94	Consensus for inclusion	**Included**
Emotional well-being assessment	0	10	89	0	8	92	0	16	81	0	11	87	Consensus for inclusion	0	6	94	0	0	100	0	5	95	0	5	95	Consensus for inclusion	**Included**
Empowerment promotion	0	11	84	0	0	100	3	19	48	1	12	77	Consensus for inclusion	0	3	97	0	9	91	0	18	82	0	7	93	Consensus for inclusion	**Included**
Engagement of community members with respectful perinatal care	0	17	78	0	17	83	0	13	87	0	16	81	Consensus for inclusion	0	11	87	0	9	91	0	9	86	0	11	87	Consensus for inclusion	**Included**
Enhancing interdisciplinary collaboration	0	11	89	0	8	92	0	19	81	0	13	87	Consensus for inclusion	0	9	90	0	0	100	0	5	91	0	7	91	Consensus for inclusion	**Included**
Enhancing willingness to learn	1	34	61	8	17	67	0	32	61	2	32	62	No consensus	0	37	63	0	55	45	0	64	36	0	45	55	No consensus	**Excluded**
Environmental safety management	0	10	90	0	17	83	0	6	90	0	10	90	Consensus for inclusion	0	10	89	0	9	91	0	9	86	0	10	88	Consensus for inclusion	**Merged for stackable meaning**
Environmental well-being management	0	16	84	0	17	83	0	26	71	0	18	81	Consensus for inclusion	0	21	77	0	9	91	0	23	73	0	20	78	Consensus for inclusion	**Merged for stackable meaning**
Facilitate breastfeeding continuation	0	1	96	0	0	100	0	13	84	0	4	94	Consensus for inclusion	0	0	100	0	0	100	0	0	100	0	0	100	Consensus for inclusion	**Included**

Facilitating breastfeeding initiation	0	1	98	0	0	100	0	6	94	0	2	97	Consensus for inclusion	0	0	100	0	0	100	0	0	100	0	0	100	Consensus for inclusion	**Included**
Facilitating social connections and support	5	40	51	8	17	67	3	35	58	5	37	54	No consensus	1	56	43	0	64	36	14	41	45	4	53	43	No consensus	**Excluded**
Facilitating visits by family and friends	4	26	68	0	25	75	0	32	68	2	27	69	No consensus	0	44	54	0	27	73	0	36	59	0	41	57	No consensus	**Excluded**
Facilitation of free movement in labour	0	7	90	0	0	100	3	19	71	1	10	86	Consensus for inclusion	0	1	99	0	9	91	0	18	82	0	6	94	Consensus for inclusion	**Included**
Fetal well-being assessment*§	0	1	96	0	17	83	0	10	82	0	5	92	Consensus for inclusion	0	0	100	0	0	100	0	0	100	0	0	100	Consensus for inclusion	**Included**
First stage of labour care	0	0	98	0	8	92	6	13	77	2	4	92	Consensus for inclusion	0	0	100	0	0	100	0	18	82	0	4	96	Consensus for inclusion	**Included**
Fourth stage of labour care	0	1	96	0	8	92	0	0	100	0	2	97	Consensus for inclusion	0	3	97	0	0	100	0	0	100	0	2	98	Consensus for inclusion	**Included**
Goals sharing	0	7	91	0	0	100	6	16	65	2	9	86	Consensus for inclusion	0	6	94	0	9	91	0	36	64	0	13	87	Consensus for inclusion	**Included**
Guided visualization	7	46	38	8	25	58	6	16	29	7	37	38	No consensus	6	76	19	0	64	36	5	73	23	5	74	21	No consensus	**Excluded**
Guidelines on how to prevent critical situations	0	22	73	0	17	75	0	16	77	0	20	74	Consensus for inclusion	0	9	91	0	0	100	0	5	95	0	7	93	Consensus for inclusion	**Merged for stackable meaning**
Health education	0	4	90	0	8	92	0	10	90	0	6	90	Consensus for inclusion	0	1	97	0	0	100	0	14	82	0	4	94	Consensus for inclusion	**Included**
Healthcare personnel development	0	6	94	0	17	83	0	16	84	0	10	90	Consensus for inclusion	0	3	96	0	18	82	0	14	82	0	7	91	Consensus for inclusion	**Included**
Healthy behaviour counselling	0	10	89	0	8	92	0	29	68	0	14	84	Consensus for inclusion	0	6	94	0	9	91	0	18	82	0	9	91	Consensus for inclusion	**Included**
Humanization of care	Midwifery intervention added after the Round 1													0	1	97	0	9	91	0	9	86	0	4	94	Consensus for inclusion	**Included**
Hydrotherapy/ immersion in water	2	34	48	0	25	75	6	29	29	3	32	46	No consensus	3	46	51	0	45	55	9	77	14	4	52	44	No consensus	**Excluded**

Hygiene management for the birthing person	0	5	94	8	0	92	3	10	84	2	6	91	Consensus for inclusion	0	7	93	0	9	91	0	18	82	0	10	90	Consensus for inclusion	**Included**
Identification of the birthing person and/or newborn	0	1	98	0	8	92	3	16	71	1	6	90	Consensus for inclusion	0	1	99	0	0	100	0	27	73	0	7	93	Consensus for inclusion	**Included**
Implementation of healthcare quality improvement programs	0	13	87	0	8	92	0	16	77	0	14	85	Consensus for inclusion	0	7	91	0	9	91	0	18	77	0	10	88	Consensus for inclusion	**Included**
Implementation of regulations/recommendations and guidelines for respectful perinatal care	0	6	94	0	8	92	0	19	77	0	10	90	Consensus for inclusion	0	1	97	0	0	100	0	18	77	0	5	93	Consensus for inclusion	**Included**
Implementation of social accountability for respectful perinatal care	0	10	87	0	17	83	0	10	90	0	10	87	Consensus for inclusion	0	7	91	0	9	91	0	9	86	0	8	90	Consensus for inclusion	**Included**
Incident reporting	0	11	89	0	8	92	0	19	68	0	13	84	Consensus for inclusion	0	4	94	0	0	100	0	5	91	0	4	94	Consensus for inclusion	**Included**
Infection prevention and control	0	7	91	0	17	83	0	13	84	0	10	89	Consensus for inclusion	0	0	99	0	0	100	0	0	95	0	0	98	Consensus for inclusion	**Included**
Interpretation of laboratory data	0	10	90	0	8	92	3	19	61	1	12	83	Consensus for inclusion	0	7	91	0	0	100	0	9	86	0	7	91	Consensus for inclusion	**Included**
Intravenous cannulation	1	24	72	8	17	75	6	39	29	3	27	62	No consensus	1	19	80	0	27	73	5	73	23	2	31	67	No consensus	**Excluded**
Latex use precautions	1	15	83	0	25	75	3	32	39	2	20	71	Consensus for inclusion	0	11	87	0	18	82	9	27	59	2	16	81	Consensus for inclusion	**Merged for stackable meaning**
Learning process facilitation	1	23	71	0	17	75	0	29	58	1	24	68	No consensus	1	16	83	0	27	73	0	41	59	1	22	77	Consensus for inclusion	**Merged for stackable meaning**
Management of acuity codes	0	6	91	0	17	83	0	6	90	0	7	90	Consensus for inclusion	0	1	97	0	9	91	0	0	95	0	2	96	Consensus for inclusion	**Merged for stackable meaning**
Management of cardiotocographic changes*	0	5	93	8	8	83	0	6	65	1	6	85	Consensus for inclusion	0	1	99	0	0	100	0	9	91	0	3	97	Consensus for inclusion	**Included**
Management of laboratory samples	0	20	79	0	17	83	0	16	74	0	18	78	Consensus for inclusion	0	21	77	0	9	91	0	18	77	0	19	79	Consensus for inclusion	**Included**

Management of newborn bowel movements	0	13	84	0	8	92	0	32	52	0	18	77	Consensus for inclusion	0	10	90	0	0	100	0	36	64	0	15	85	Consensus for inclusion	**Included**
Management of newborn nutrition	0	1	98	0	0	100	3	0	94	1	1	97	Consensus for inclusion	0	1	99	0	0	100	0	9	91	0	3	97	Consensus for inclusion	**Included**
Management of nutrition in pregnancy/childbirth/postpartum	1	16	82	0	0	100	0	32	65	1	18	79	Consensus for inclusion	0	9	91	0	9	91	0	41	59	0	16	84	Consensus for inclusion	**Included**
Management of supplies	2	29	66	8	42	50	3	16	71	3	27	66	No consensus	1	47	50	0	82	18	0	32	64	1	48	50	No consensus	**Excluded**
Management of technical equipment and devices	0	35	62	0	33	67	0	29	65	0	34	63	No consensus	1	31	66	0	9	91	0	36	59	1	30	67	Consensus for inclusion	**Included**
Management of the birthing person's bowel movements	0	22	77	0	17	83	0	45	42	0	27	69	No consensus	0	33	67	0	9	91	0	50	50	0	34	66	Consensus for inclusion	**Included**
Management of the birthing person's micturition	1	12	85	0	8	92	0	39	48	1	18	77	Consensus for inclusion	0	14	86	0	9	91	0	50	50	0	21	79	Consensus for inclusion	**Included**
Management of the environment	0	23	77	0	17	83	0	19	77	0	22	78	Consensus for inclusion	0	21	77	0	9	91	5	18	73	1	19	78	Consensus for inclusion	**Included**
Management of the newborn's micturition	0	12	85	0	8	92	0	32	52	0	17	78	Consensus for inclusion	0	14	86	0	0	100	0	36	64	0	17	83	Consensus for inclusion	**Included**
Management of vaccinations	1	15	79	0	17	83	0	6	94	1	13	83	Consensus for inclusion	1	11	86	0	18	82	0	14	82	1	13	84	Consensus for inclusion	**Included**
Massage	0	50	45	0	25	75	0	58	39	0	50	46	No consensus	3	54	43	0	55	45	0	68	32	2	57	41	No consensus	**Excluded**
Maternal-fetal monitoring in labour	0	5	93	8	0	92	0	13	81	1	6	90	Consensus for inclusion	0	3	97	0	9	91	0	14	86	0	6	94	Consensus for inclusion	**Included**
Medication management	0	12	87	0	17	83	0	13	84	0	13	86	Consensus for inclusion	0	6	93	0	0	100	0	18	77	0	8	90	Consensus for inclusion	**Included**
Mentorship and supervision of students	0	13	87	0	17	83	0	23	55	0	16	78	Consensus for inclusion	0	7	91	0	9	91	0	14	82	0	9	89	Consensus for inclusion	**Included**

Midwife-led continuity of carer	0	6	94	0	8	92	0	23	71	0	10	88	Consensus for inclusion	0	4	94	0	0	100	0	18	77	0	7	91	Consensus for inclusion	**Included**
Midwifery clinical consultation	0	13	85	0	8	92	3	29	68	1	17	82	Consensus for inclusion	0	11	87	0	9	91	0	14	82	0	12	86	Consensus for inclusion	**Included**
Midwifery clinical handover	0	4	96	0	17	83	3	13	68	1	7	88	Consensus for inclusion	1	1	96	0	9	91	0	5	91	1	3	94	Consensus for inclusion	**Included**
Monitoring of health policy	0	24	68	0	8	92	0	16	81	0	21	74	Consensus for inclusion	1	30	67	0	9	91	0	18	77	1	25	72	Consensus for inclusion	**Included**
Monitoring of term pregnancy*	0	6	91	8	0	92	0	16	77	1	8	88	Consensus for inclusion	0	3	97	0	9	91	0	0	100	0	3	97	Consensus for inclusion	**Included**
Monitoring of vital signs	0	5	94	0	8	92	0	13	84	0	7	91	Consensus for inclusion	0	4	96	0	0	100	0	0	100	0	3	97	Consensus for inclusion	**Included**
Multidisciplinary clinical consultation	0	13	87	0	8	92	3	26	58	1	16	80	Consensus for inclusion	0	13	86	0	9	91	0	23	73	0	15	83	Consensus for inclusion	**Included**
Negotiation of care with birthing person	4	21	70	8	0	83	0	23	61	3	19	69	No consensus	1	29	70	9	9	82	5	50	45	3	31	66	No consensus	**Excluded**
Newborn bowel movements counselling	0	13	84	0	8	92	0	19	58	0	14	78	Consensus for inclusion	0	11	89	0	0	100	0	27	73	0	14	86	Consensus for inclusion	**Included**
Newborn care counselling	0	2	96	0	8	92	3	6	90	1	4	94	Consensus for inclusion	0	0	100	0	0	100	0	5	95	0	1	99	Consensus for inclusion	**Included**
Newborn hygiene management	0	4	94	0	8	92	0	10	87	0	6	92	Consensus for inclusion	0	6	94	0	9	91	0	18	82	0	9	91	Consensus for inclusion	**Included**
Newborn nutrition counselling	0	2	96	0	8	92	0	3	94	0	3	95	Consensus for inclusion	0	0	100	0	9	91	0	5	95	0	2	98	Consensus for inclusion	**Included**
Newborn physical examination	0	5	93	0	0	100	3	3	90	1	4	93	Consensus for inclusion	0	4	96	0	0	100	0	9	91	0	5	95	Consensus for inclusion	**Included**
Newborn prophylaxis	0	11	87	0	8	92	0	6	87	0	10	87	Consensus for inclusion	0	10	90	0	9	91	0	9	91	0	10	90	Consensus for inclusion	**Included**

Newborn urinary counselling	0	10	88	0	8	92	0	26	61	0	14	82	Consensus for inclusion	0	10	90	0	0	100	0	32	68	0	14	86	Consensus for inclusion	**Included**
Non-pharmacological treatment prescription	0	33	66	0	8	92	0	32	58	0	30	66	Consensus for inclusion	0	26	73	0	18	82	0	36	59	0	27	71	Consensus for inclusion	**Included**
Nutrition counselling for the birthing person	0	16	83	0	17	83	3	42	52	1	22	75	Consensus for inclusion	0	14	86	0	9	91	0	41	59	0	19	81	Consensus for inclusion	**Included**
Obstetric triage	0	1	95	0	8	92	0	6	90	0	3	94	Consensus for inclusion	0	0	100	0	0	100	0	5	95	0	1	99	Consensus for inclusion	**Included**
Orientation of healthcare personnel	0	10	90	0	25	75	0	13	68	0	12	83	Consensus for inclusion	0	1	97	0	18	82	0	14	82	0	6	92	Consensus for inclusion	**Included**
Pain management	0	7	91	0	8	92	0	10	81	0	8	89	Consensus for inclusion	0	0	100	0	0	100	0	14	86	0	3	97	Consensus for inclusion	**Included**
Parental role promotion	0	11	84	0	17	83	3	13	74	1	12	82	Consensus for inclusion	0	1	99	0	0	100	0	14	86	0	4	96	Consensus for inclusion	**Included**
Partograph	1	13	84	0	8	92	0	16	71	1	14	82	Consensus for inclusion	0	11	87	0	18	82	0	14	82	0	13	85	Consensus for inclusion	**Merged for stackable meaning**
Perineal care	0	9	90	0	0	100	0	26	71	0	12	86	Consensus for inclusion	0	3	97	0	0	100	0	9	91	0	4	96	Consensus for inclusion	**Included**
Perineal repair	0	12	84	0	8	92	0	13	77	0	12	83	Consensus for inclusion	0	6	94	0	0	100	0	0	100	0	4	96	Consensus for inclusion	**Included**
Perineal tear care	0	5	94	0	0	100	0	13	84	0	6	92	Consensus for inclusion	0	0	100	0	0	100	0	5	95	0	1	99	Consensus for inclusion	**Included**
Personalized care	0	5	94	0	0	100	0	26	74	0	10	90	Consensus for inclusion	0	3	96	0	0	100	0	9	86	0	4	94	Consensus for inclusion	**Included**
Physical examination of the birthing person	0	5	94	0	0	100	0	16	81	0	7	91	Consensus for inclusion	0	0	100	0	0	100	0	9	91	0	2	98	Consensus for inclusion	**Included**
Point-of-Care Ultrasound (POCUS)	0	38	56	17	25	58	0	23	48	2	33	54	No consensus	0	50	50	0	36	64	5	36	59	1	46	53	No consensus	**Excluded**

Postnatal care	0	0	99	0	0	100	0	13	84	0	3	95	Consensus for inclusion	0	0	100	0	0	100	0	18	82	0	4	96	Consensus for inclusion	**Included**
Postpartum haemorrhage prevention	0	5	93	0	8	92	0	6	87	0	6	91	Consensus for inclusion	0	0	100	0	9	91	0	0	100	0	1	99	Consensus for inclusion	**Included**
Prenatal diagnosis counselling	0	13	85	0	8	92	0	13	84	0	13	86	Consensus for inclusion	0	9	91	0	9	91	0	9	91	0	9	91	Consensus for inclusion	**Included**
Presence	0	5	93	0	8	83	0	10	87	0	6	90	Consensus for inclusion	0	3	97	0	9	91	0	0	100	0	3	97	Consensus for inclusion	**Included**
Prevention of falls	0	26	74	0	17	83	3	32	58	1	26	71	Consensus for inclusion	0	13	86	0	0	100	0	23	73	0	14	84	Consensus for inclusion	**Included**
Promoting health awareness at community level	0	5	93	0	0	100	0	13	81	0	6	90	Consensus for inclusion	0	6	93	0	0	100	0	14	82	0	7	91	Consensus for inclusion	**Included**
Promotion of cord blood donation	2	33	63	17	25	58	0	19	77	3	29	66	No consenso	0	41	57	18	27	55	0	36	59	2	39	57	No consensus	**Excluded**
Promotion of free movement and positions during labour and birth	0	9	90	0	0	100	3	13	77	1	9	88	Consensus for inclusion	0	1	99	0	9	91	0	14	86	0	5	95	Consensus for inclusion	**Included**
Promotion of health literacy	0	29	65	0	17	83	0	29	52	0	28	63	No consensus	0	24	76	0	9	91	0	55	45	0	29	71	Consensus for inclusion	**Included**
Promotion of parental-infant bonding	0	4	95	0	8	92	3	13	81	1	6	91	Consensus for inclusion	0	1	99	0	0	100	0	5	95	0	2	98	Consensus for inclusion	**Included**
Promotion of physical activity/exercise	0	18	78	0	8	92	3	42	55	1	23	74	Consensus for inclusion	0	14	84	0	9	91	0	50	45	0	21	77	Consensus for inclusion	**Included**
Promotion of physiological processes during labour and birth	0	4	91	0	17	83	0	10	87	0	6	90	Consensus for inclusion	0	3	96	0	18	82	0	18	77	0	8	90	Consensus for inclusion	**Included**
Promotion of self-awareness	0	21	77	0	17	75	0	29	68	0	22	74	Consensus for inclusion	0	17	83	0	9	91	0	27	73	0	18	82	Consensus for inclusion	**Included**
Promotion of self-care	0	17	80	0	0	100	3	23	71	1	17	80	Consensus for inclusion	0	3	97	0	9	91	0	14	86	0	6	94	Consensus for inclusion	**Included**

Promotion of self-efficacy	0	22	72	0	17	83	0	32	42	0	24	66	No consensus	0	19	81	0	0	100	0	41	59	0	21	79	Consensus for inclusion	**Included**
Protecting the rights of birthing people and newborns	0	5	95	0	17	83	0	6	94	0	6	94	Consensus for inclusion	0	3	96	0	0	100	0	0	95	0	2	96	Consensus for inclusion	**Included**
Provide guidance on navigating the healthcare service	0	35	61	0	33	67	0	42	52	0	37	59	No consensus	0	40	59	0	55	45	0	41	55	0	42	56	No consensus	**Excluded**
Quality monitoring	1	17	82	0	17	83	0	39	61	1	22	77	Consensus for inclusion	0	9	90	0	0	100	0	14	82	0	9	89	Consensus for inclusion	**Included**
Risk assessment for the birthing person	0	4	96	0	8	92	3	0	97	1	3	96	Consensus for inclusion	0	0	99	0	9	91	0	0	95	0	1	97	Consensus for inclusion	**Included**
Risk assessment for the fetus/newborn	0	2	98	0	8	92	3	0	97	1	2	97	Consensus for inclusion	0	0	99	0	9	91	0	5	91	0	2	96	Consensus for inclusion	**Included**
Risk identification	0	2	98	0	8	92	0	26	65	0	9	89	Consensus for inclusion	0	1	97	0	0	100	0	5	91	0	2	96	Consensus for inclusion	**Included**
Role empowerment	1	32	65	8	8	75	0	19	68	2	26	66	No consensus	0	23	77	0	9	91	0	45	55	0	26	74	Consensus for inclusion	**Merged for stackable meaning**
Rooming-in	0	5	95	0	0	100	3	26	65	1	10	88	Consensus for inclusion	0	3	96	0	0	100	0	14	82	0	5	93	Consensus for inclusion	**Included**
Screening	0	6	88	0	8	92	0	3	97	0	6	90	Consensus for inclusion	0	1	97	0	9	91	0	5	91	0	3	95	Consensus for inclusion	**Included**
Second stage of labour care	0	0	98	0	8	92	0	13	84	0	4	94	Consensus for inclusion	0	0	100	0	0	100	0	9	91	0	2	98	Consensus for inclusion	**Included**
Sexual and reproductive health counselling*	0	28	71	0	25	75	3	32	61	1	29	69	No consensus	0	6	94	0	0	100	0	45	55	0	14	86	Consensus for inclusion	**Included**
Signs and symptoms counselling	1	10	88	0	0	100	3	32	61	2	14	82	Consensus for inclusion	0	13	87	0	0	100	5	23	73	1	14	85	Consensus for inclusion	**Included**
Skin to skin	0	9	90	0	0	100	3	13	84	1	9	90	Consensus for inclusion	0	1	99	0	0	100	0	18	82	0	5	95	Consensus for inclusion	**Included**

Supervision of healthcare personnel	0	18	79	0	33	67	0	16	81	0	19	78	Consensus for inclusion	0	7	91	0	18	82	0	9	86	0	9	89	Consensus for inclusion	**Included**
Support for behaviour change	0	28	65	0	8	75	0	32	65	0	27	66	No consensus	0	34	66	0	36	64	5	18	77	1	31	68	No consensus	**Excluded**
Support for those providing respectful perinatal care	1	11	87	0	17	83	0	13	87	1	12	86	Consensus for inclusion	0	4	94	0	0	100	0	14	82	0	6	92	Consensus for inclusion	**Included**
Support to manage behaviour	0	23	71	0	8	75	0	26	68	0	22	70	Consensus for inclusion	0	20	80	0	9	91	5	18	77	1	18	81	Consensus for inclusion	**Included**
Supporting newborn adaptation	0	0	98	0	8	92	0	3	94	0	2	96	Consensus for inclusion	0	0	100	0	0	100	0	0	100	0	0	100	Consensus for inclusion	**Included**
Team cooperation	0	2	96	0	8	92	6	19	65	2	7	88	Consensus for inclusion	0	0	99	0	0	100	0	18	77	0	4	94	Consensus for inclusion	**Included**
Telehealth	4	50	35	8	17	75	0	32	45	3	42	42	No consensus	4	73	23	0	18	82	5	77	18	4	68	28	No consensus	**Excluded**
Telephone consultations	6	49	44	8	17	75	3	48	45	6	46	47	No consensus	3	57	40	0	18	82	0	77	23	2	57	41	No consensus	**Excluded**
Telephone follow-up	5	54	39	0	17	83	3	39	48	4	46	46	No consensus	3	69	29	0	27	73	0	45	55	2	59	39	No consensus	**Excluded**
Telephone triage	6	29	57	0	8	92	10	26	52	6	26	59	Consensus for inclusion	3	44	53	0	18	82	0	55	45	2	44	54	No consensus	**Excluded**
Third stage of labour care	0	0	98	0	8	92	0	0	97	0	1	97	Consensus for inclusion	0	1	99	0	0	100	0	0	100	0	1	99	Consensus for inclusion	**Included**
Training healthcare professionals on respectful perinatal care	0	9	91	0	0	100	0	0	97	0	6	94	Consensus for inclusion	0	3	96	0	0	100	0	0	95	0	2	96	Consensus for inclusion	**Included**
Transfer within the facility	4	37	52	8	25	67	3	35	42	4	35	51	No consensus	0	46	53	0	73	27	5	32	59	1	46	51	No consensus	**Excluded**
Umbilical cord care	0	12	87	0	0	100	0	10	87	0	10	88	Consensus for inclusion	0	9	91	0	9	91	0	9	91	0	9	91	Consensus for inclusion	**Included**
Venous blood sampling	0	29	67	0	17	83	0	26	52	0	27	65	No consensus	0	21	79	0	27	73	5	32	64	1	24	75	Consensus for inclusion	**Excluded for consistency**

Welcome upon arrival at the facility	1	11	87	0	33	67	0	19	81	1	15	83	Consensus for inclusion	0	3	97	0	0	100	0	18	82	0	6	94	Consensus for inclusion	**Included**

*Midwifery interventions with wording modifications proposed by participants and authors in Round 1

§Average score in Round 1 across two midwifery interventions (i.e., “Fetal well-being assessment: FHR” and “Fetal well-being assessment: FM”) both achieving consensus for inclusion and after Round 1 for consistency
