# Supplementary material for: Development and Validation of the Midwifery Interventions Classification for a Salutogenic Approach to Maternity Care: A Delphi Study
Source: Healthcare (Basel). 2024 Nov 8;12(22):2228. doi: 10.3390/healthcare12222228 (PMC11594468; doi:10.3390/healthcare12222228)
Supplement: Supplementary file 1 [file healthcare-12-02228-s001.zip › Table S5.pdf]

Table S5. Demographics characteristics of non-responders in the second round Delphi

| Characteristics | Panel of Midwifes (n=12) | Panel of Researchers (n=1) | Panel of Maternity Users services (n=9) |
|---|---|---|---|
| Gender, n (%) | | | |
| Female | 11 (91.6) | 1 (100) | 9 (100) |
| Male | 1 (8.4) | 0 | 0 |
| Civil status, n (%) | | | |
| Married | 5 (41.7) | 0 | 4 (44.5) |
| Not married | 7 (58.3) | 1 (100) | 5 (55.5) |
| Education, n (%) | | | |
| Secondary Education | 1 (8.4) | 0 | 0 |
| Bachelor’s Degree | 8 (66.6) | 1 (100) | 4 (44.4) |
| Postgraduate Education | 3 (25) | 0 | 5 (55.6) |
| Age (years) (M±SD) | 39.6±9.5 | na | 34.3±4.0 |
