# Supplementary material for: Development and Validation of the Midwifery Interventions Classification for a Salutogenic Approach to Maternity Care: A Delphi Study
Source: Healthcare (Basel). 2024 Nov 8;12(22):2228. doi: 10.3390/healthcare12222228 (PMC11594468; doi:10.3390/healthcare12222228)
Supplement: Supplementary file 1 [file healthcare-12-02228-s001.zip › Table S6.pdf]

Table S6: Intraclass coefficient correlation (ICC)

Item	Service users Panel		Researchers Panel		Midwives Panel	
	ICC	95% CI	ICC	95% CI	ICC	95% CI
Item_1	0.99	.970-.995	1	1.000-1.000	1	1.000-1.000
Item_2	0.91	.746-.746	1	1.000-1.000	1	1.000-1.000
Item_3	0.95	.851-.983	1	1.000-1.000	1	1.000-1.000
Item_4	0.99	.976-.996	1	1.000-1.000	1	1.000-1.000
Item_5	0.99	.978-.996	1	1.000-1.000	1	1.000-1.000
Item_6	0.90	.750-.957	1	1.000-1.000	1	1.000-1.000
Item_7	1.00	1.00-1.00	1	1.000-1.000	1	1.000-1.000
Item_8	1.00	1.00-1.00	1	1.000-1.000	1	1.000-1.000
Item_9	0.95	.869-.978	1	1.000-1.000	1	1.000-1.000
Item_10	0.95	.887-.981	1	1.000-1.000	1	1.000-1.000
Item_11	0.95	.872-.979	1	1.000-1.000	1	1.000-1.000
Item_12	0.93	.829-.971	1	1.000-1.000	1	1.000-1.000
Item_13	0.92	.813-.969	1	1.000-1.000	1	1.000-1.000
Item_14	0.81	.524-.922	1	1.000-1.000	1	1.000-1.000
Item_15	1.00	1.00-1.00	1	1.000-1.000	1	1.000-1.000
Item_16	0.78	.455-.910	1	1.000-1.000	1	1.000-1.000
Item_17	0.92	.804-.968	1	1.000-1.000	1	1.000-1.000
Item_18	0.99	.971-.996	1	1.000-1.000	1	1.000-1.000
Item_19	1.00	1.00-1.00	1	1.000-1.000	1	1.000-1.000
Item_20	0.98	.945-.992	1	1.000-1.000	1	1.000-1.000
Item_21	0.85	.622-.938	1	1.000-1.000	1	1.000-1.000
Item_22	0.97	.902-.987	1	1.000-1.000	1	1.000-1.000
Item_23	0.92	.799-.969	1	1.000-1.000	1	1.000-1.000
Item_24	0.93	.832-.974	1	1.000-1.000	1	1.000-1.000
Item_25	1.00	1.00-1.00	1	1.000-1.000	1	1.000-1.000
Item_26	0.93	.839-.972	1	1.000-1.000	1	1.000-1.000
Item_27	0.94	.850-.975	1	1.000-1.000	1	1.000-1.000
Item_28	0.99	.985-.998	1	1.000-1.000	1	1.000-1.000
Item_29	0.99	.984-.997	1	1.000-1.000	1	1.000-1.000
Item_30	0.99	.978-.997	1	1.000-1.000	1	1.000-1.000
Item_31	0.94	.832-.975	1	1.000-1.000	1	1.000-1.000
Item_32	0.99	.982-.997	1	1.000-1.000	1	1.000-1.000
Item_33	0.92	.783-.970	1	1.000-1.000	1	1.000-1.000
Item_34	0.97	.924-.988	1	1.000-1.000	1	1.000-1.000
Item_35	0.98	.954-.992	1	1.000-1.000	1	1.000-1.000
Item_36	0.96	.911-.985	1	1.000-1.000	1	1.000-1.000
Item_37	0.98	.961-.994	1	1.000-1.000	1	1.000-1.000
Item_38	0.96	.903-.983	1	1.000-1.000	1	1.000-1.000
Item_39	0.99	.968-.995	1	1.000-1.000	1	1.000-1.000
Item_40	1.00	1.00-1.00	1	1.000-1.000	1	1.000-1.000
Item_41	1.00	1.00-1.00	1	1.000-1.000	1	1.000-1.000
Item_42	1.00	1.00-1.00	1	1.000-1.000	1	1.000-1.000

Table S6: Intraclass coefficient correlation (ICC)

Item_43	0.99	.985-.998	1	1.000-1.000	1	1.000-1.000
Item_44	0.92	.783-.968	1	1.000-1.000	1	1.000-1.000
Item_45	0.99	.985-.998	1	1.000-1.000	1	1.000-1.000
Item_46	0.93	.820-.973	1	1.000-1.000	1	1.000-1.000
Item_47	0.98	.943-.991	1	1.000-1.000	1	1.000-1.000
Item_48	0.97	.932-.989	1	1.000-1.000	1	1.000-1.000
Item_49	1.00	1.00-1.00	1	1.000-1.000	1	1.000-1.000
Item_50	1.00	.988-.998	1	1.000-1.000	1	1.000-1.000
Item_51	1.00	1.00-1.00	1	1.000-1.000	1	1.000-1.000
Item_52	0.98	.944-.993	1	1.000-1.000	1	1.000-1.000
Item_53	1.00	1.00-1.00	1	1.000-1.000	1	1.000-1.000
Item_54	0.99	.982-.997	1	1.000-1.000	1	1.000-1.000
Item_55	1.00	1.00-1.00	1	1.000-1.000	1	1.000-1.000
Item_56	1.00	1.00-1.00	1	1.000-1.000	1	1.000-1.000
Item_57	0.97	.933-.989	1	1.000-1.000	1	1.000-1.000
Item_58	0.95	.886-.981	1	1.000-1.000	1	1.000-1.000
Item_59	0.98	.945-.993	1	1.000-1.000	1	1.000-1.000
Item_60	0.94	.859-.977	1	1.000-1.000	1	1.000-1.000
Item_61	0.96	.846-.987	1	1.000-1.000	1	1.000-1.000
Item_62	0.98	.931-.992	1	1.000-1.000	1	1.000-1.000
Item_63	0.98	.936-.992	1	1.000-1.000	1	1.000-1.000
Item_64	0.98	.945-.990	1	1.000-1.000	1	1.000-1.000
Item_65	0.98	.953-.992	1	1.000-1.000	1	1.000-1.000
Item_66	0.96	.895-.983	1	1.000-1.000	1	1.000-1.000
Item_67	0.94	.848-.975	1	1.000-1.000	1	1.000-1.000
Item_68	0.84	.610-.936	1	1.000-1.000	1	1.000-1.000
Item_69	0.98	.940-.990	1	1.000-1.000	1	1.000-1.000
Item_70	1.00	1.00-1.00	1	1.000-1.000	1	1.000-1.000
Item_71	1.00	1.00-1.00	1	1.000-1.000	1	1.000-1.000
Item_72	1.00	1.00-1.00	1	1.000-1.000	1	1.000-1.000
Item_73	0.98	.960-.993	1	1.000-1.000	1	1.000-1.000
Item_74	0.86	.651-.943	1	1.000-1.000	1	1.000-1.000
Item_75	0.80	.500-.922	1	1.000-1.000	1	1.000-1.000
Item_76	0.96	.893-.983	1	1.000-1.000	1	1.000-1.000
Item_77	0.95	.881-.982	1	1.000-1.000	1	1.000-1.000
Item_78	1.00	.988-.998	1	1.000-1.000	1	1.000-1.000
Item_79	0.96	.873-.985	1	1.000-1.000	1	1.000-1.000
Item_80	0.98	.952-.992	1	1.000-1.000	1	1.000-1.000
Item_81	0.96	.898-.984	1	1.000-1.000	1	1.000-1.000
Item_82	1.00	1.00-1.00	1	1.000-1.000	1	1.000-1.000
Item_83	1.00	1.00-1.00	1	1.000-1.000	1	1.000-1.000
Item_84	1.00	1.00-1.00	1	1.000-1.000	1	1.000-1.000
Item_85	0.99	.978-.997	1	1.000-1.000	1	1.000-1.000
Item_86	0.99	.980-.997	1	1.000-1.000	1	1.000-1.000

Table S6: Intraclass coefficient correlation (ICC)

Item_87	0.96	.883-.987	1	1.000-1.000	1	1.000-1.000
Item_88	0.96	.896-.985	1	1.000-1.000	1	1.000-1.000
Item_89	0.97	.931-.989	1	1.000-1.000	1	1.000-1.000
Item_90	0.93	.838-.973	1	1.000-1.000	1	1.000-1.000
Item_91	0.94	.840-.840	1	1.000-1.000	1	1.000-1.000
Item_92	0.98	.946-.991	1	1.000-1.000	1	1.000-1.000
Item_93	1.00	1.00-1.00	1	1.000-1.000	1	1.000-1.000
Item_94	0.96	.907-.985	1	1.000-1.000	1	1.000-1.000
Item_95	0.94	.842-.978	1	1.000-1.000	1	1.000-1.000
Item_96	1.00	1.00-1.00	1	1.000-1.000	1	1.000-1.000
Item_97	0.93	.826-.973	1	1.000-1.000	1	1.000-1.000
Item_98	0.97	.924-.989	1	1.000-1.000	1	1.000-1.000
Item_99	0.99	.979-.998	1	1.000-1.000	1	1.000-1.000
Item_100	1.00	1.00-1.00	1	1.000-1.000	1	1.000-1.000
Item_101	0.98	.939-.989	1	1.000-1.000	1	1.000-1.000
Item_102	0.92	.777-.973	1	1.000-1.000	1	1.000-1.000
Item_103	0.95	.884-.981	1	1.000-1.000	1	1.000-1.000
Item_104	1.00	1.00-1.00	1	1.000-1.000	1	1.000-1.000
Item_105	1.00	1.00-1.00	1	1.000-1.000	1	1.000-1.000
Item_106	0.96	.909-.984	1	1.000-1.000	1	1.000-1.000
Item_107	0.95	.880-.982	1	1.000-1.000	1	1.000-1.000
Item_108	0.92	.784-.966	1	1.000-1.000	1	1.000-1.000
Item_109	1.00	1.00-1.00	1	1.000-1.000	1	1.000-1.000
Item_110	0.96	.892-.982	1	1.000-1.000	1	1.000-1.000
Item_111	1.00	1.00-1.00	1	1.000-1.000	1	1.000-1.000
Item_112	1.00	1.00-1.00	1	1.000-1.000	1	1.000-1.000
Item_113	0.95	.889-.981	1	1.000-1.000	1	1.000-1.000
Item_114	0.94	.846-.975	1	1.000-1.000	1	1.000-1.000
Item_115	1.00	1.00-1.00	1	1.000-1.000	1	1.000-1.000
Item_116	1.00	1.00-1.00	1	1.000-1.000	1	1.000-1.000
Item_117	0.99	.982-.997	1	1.000-1.000	1	1.000-1.000
Item_118	0.97	.936-.989	1	1.000-1.000	1	1.000-1.000
Item_119	0.99	.986-.998	1	1.000-1.000	1	1.000-1.000
Item_120	1.00	1.00-1.00	1	1.000-1.000	1	1.000-1.000
Item_121	0.99	.972-.995	1	1.000-1.000	1	1.000-1.000
Item_122	0.99	.985-.998	1	1.000-1.000	1	1.000-1.000
Item_123	1.00	1.00-1.00	1	1.000-1.000	1	1.000-1.000
Item_124	0.98	.944-.992	1	1.000-1.000	1	1.000-1.000
Item_125	1.00	1.00-1.00	1	1.000-1.000	1	1.000-1.000
Item_126	1.00	1.00-1.00	1	1.000-1.000	1	1.000-1.000
Item_127	0.99	.984-.997	1	1.000-1.000	1	1.000-1.001
Item_128	1.00	1.00-1.00	1	1.000-1.000	1	1.000-1.000
Item_129	1.00	1.00-1.00	1	1.000-1.000	1	1.000-1.000
Item_130	1.00	.990-.999	1	1.000-1.000	1	1.000-1.000

Table S6: Intraclass coefficient correlation (ICC)

Item_131	0.99	.971-.995	1	1.000-1.000	1	1.000-1.000
Item_132	0.99	.975-.996	1	1.000-1.000	1	1.000-1.000
Item_133	0.94	.851-.974	1	1.000-1.000	1	1.000-1.000
Item_134	0.99	.965-.994	1	1.000-1.000	1	1.000-1.000
Item_135	0.96	.913-.985	1	1.000-1.000	1	1.000-1.000
Item_136	1.00	1.00-1.00	1	1.000-1.000	1	1.000-1.000
Item_137	0.99	.962-.994	1	1.000-1.000	1	1.000-1.000
Item_138	0.99	.984-.997	1	1.000-1.000	1	1.000-1.000
Item_139	0.98	.961-.994	1	1.000-1.000	1	1.000-1.000
Item_140	0.99	.964-.994	1	1.000-1.000	1	1.000-1.000
Item_141	0.99	.962-.994	1	1.000-1.000	1	1.000-1.000
Item_142	1.00	1.00-1.00	1	1.000-1.000	1	1.000-1.000
Item_143	0.99	.984-.997	1	1.000-1.000	1	1.000-1.000
Item_144	0.95	.885-.980	1	1.000-1.000	1	1.000-1.000
Item_145	0.99	.982-.997	1	1.000-1.000	1	1.000-1.000
Item_146	1.00	.989-.998	1	1.000-1.000	1	1.000-1.000
Item_147	0.96	.911-.985	1	1.000-1.000	1	1.000-1.000
Item_148	0.97	.922-.989	1	1.000-1.000	1	1.000-1.000
Item_149	0.98	.947-.993	1	1.000-1.000	1	1.000-1.000
Item_150	1.00	1.00-1.00	1	1.000-1.000	1	1.000-1.000
Item_151	0.99	.986-.998	1	1.000-1.000	1	1.000-1.000
Item_152	0.99	.974-.995	1	1.000-1.000	1	1.000-1.000
Item_153	0.89	.728-.953	1	1.000-1.000	1	1.000-1.000
Item_154	0.94	.865-.977	1	1.000-1.000	1	1.000-1.000
Item_155	0.96	.894-.982	1	1.000-1.000	1	1.000-1.000
Item_156	0.92	.797-.965	1	1.000-1.000	1	1.000-1.000
Item_157	0.95	.884-.980	1	1.000-1.000	1	1.000-1.000
Item_158	0.99	.984-.997	1	1.000-1.000	1	1.000-1.000
Item_159	0.97	.934-.989	1	1.000-1.000	1	1.000-1.000
Item_160	0.90	.742-.958	1	1.000-1.000	1	1.000-1.000
Item_161	0.91	.773-.963	1	1.000-1.000	1	1.000-1.000
Item_162	0.99	.984-.997	1	1.000-1.000	1	1.000-1.000
Item_163	0.96	.895-.982	1	1.000-1.000	1	1.000-1.000
Item_164	0.98	.948-.992	1	1.000-1.000	1	1.000-1.000
Item_165	1.00	1.00-1.00	1	1.000-1.000	1	1.000-1.000
Item_166	na	na	na	na	na	na

Python syntax

```
# Apri un file di testo per scrivere la sintassi generata

with open("sintassi_ICC_spss.txt", "w") as file:

    # Intestazione iniziale

    file.write("SORT CASES BY category.\n")
```

Table S6: Intraclass coefficient correlation (ICC)

```
file.write("SPLIT FILE BY category.\n\n")


# Ciclo per generare ogni comando RELIABILITY per ciascun item
for i in range(1, 166):
    sintassi = (
        f"RELIABILITY /VARIABLES=Round1_item{i} Round2_item{i} "
        "/SCALE('ALL VARIABLES') ALL /MODEL=ALPHA /STATISTICS=DESCRIPTIVE "
        "/ICC=MODEL(MIXED) TYPE(CONSISTENCY) CIN=95 TESTVAL=0.\n"
    )
    file.write(sintassi)


# Chiusura del comando SPLIT FILE
file.write("\nSPLIT FILE OFF.\n")
```
