# Supplementary material for: Development and Validation of the Midwifery Interventions Classification for a Salutogenic Approach to Maternity Care: A Delphi Study
Source: Healthcare (Basel). 2024 Nov 8;12(22):2228. doi: 10.3390/healthcare12222228 (PMC11594468; doi:10.3390/healthcare12222228)
Supplement: Supplementary file 1 [file healthcare-12-02228-s001.zip › Table S7.pdf]

Table S7. Content Validity Ratio (CVR) for the 2° Round Delphi (n= 103 responders)

	Midwives Panel (n=70)			Researchers Panel (n=11)			Service Users Panel (n=22)			Decision
	Ne	CVR	Interpretation	Ne	CVR	Interpretation	Ne	CVR	Interpretation	Interpretation
Item1	68	0.94	Essential	11	1.00	Essential	18	0.64	Essential	Y
Item2	12	-0.66	Essential	4	-0.27	Not Essential	14	0.27	Not Essential	N
Item3	20	-0.43	Essential	3	-0.45	Essential	3	-0.73	Not Essential	N
Item4	69	0.97	Essential	11	1.00	Essential	20	0.82	Essential	Y
Item5	64	0.83	Essential	10	0.82	Essential	13	0.18	Not Essential	Y
Item6	70	1.00	Essential	11	1.00	Essential	22	1.00	Essential	Y
Item7	70	1.00	Essential	11	1.00	Essential	22	1.00	Essential	Y
Item8	69	0.97	Essential	11	1.00	Essential	22	1.00	Essential	Y
Item9	70	1.00	Essential	11	1.00	Essential	18	0.64	Essential	Y
Item10	70	1.00	Essential	11	1.00	Essential	20	0.82	Essential	Y
Item11	69	0.97	Essential	11	1.00	Essential	22	1.00	Essential	Y
Item12	68	0.94	Essential	11	1.00	Essential	22	1.00	Essential	Y
Item13	70	1.00	Essential	11	1.00	Essential	22	1.00	Essential	Y
Item14	62	0.77	Essential	9	0.64	Essential	14	0.27	Not Essential	Y
Item15	70	1.00	Essential	11	1.00	Essential	22	1.00	Essential	Y
Item16	70	1.00	Essential	11	1.00	Essential	18	0.64	Essential	Y
Item17	70	1.00	Essential	11	1.00	Essential	18	0.64	Essential	Y
Item18	69	0.97	Essential	11	1.00	Essential	20	0.82	Essential	Y
Item19	60	0.71	Essential	10	0.82	Essential	17	0.55	Essential	Y
Item20	66	0.89	Essential	10	0.82	Essential	14	0.27	Not Essential	Y
Item21	28	-0.20	Not Essential	9	0.64	Essential	5	-0.55	Not Essential	N
Item22	69	0.97	Essential	11	1.00	Essential	18	0.64	Essential	Y
Item23	49	0.40	Essential	9	0.64	Essential	10	-0.09	Not Essential	N
Item24	70	1.00	Essential	10	0.82	Essential	15	0.36	Not Essential	Y
Item25	67	0.91	Essential	11	1.00	Essential	17	0.55	Essential	Y
Item26	66	0.89	Essential	11	1.00	Essential	12	0.09	Not Essential	Y
Item27	70	1.00	Essential	11	1.00	Essential	21	0.91	Essential	Y
Item28	70	1.00	Essential	10	0.82	Essential	21	0.91	Essential	Y
Item29	66	0.89	Essential	10	0.82	Essential	18	0.64	Essential	Y
Item30	64	0.83	Essential	10	0.82	Essential	20	0.82	Essential	Y
Item31	62	0.77	Essential	11	1.00	Essential	16	0.45	Essential	Y
Item32	47	0.34	Essential	10	0.82	Essential	10	-0.09	Not Essential	Y
Item33	63	0.80	Essential	11	1.00	Essential	15	0.36	Not Essential	Y
Item34	60	0.71	Essential	10	0.82	Essential	13	0.18	Not Essential	Y
Item35	70	1.00	Essential	10	0.82	Essential	21	0.91	Essential	Y
Item36	60	0.71	Essential	10	0.82	Essential	13	0.18	Not Essential	Y
Item37	60	0.71	Essential	10	0.82	Essential	7	-0.36	Essential	Y
Item38	61	0.74	Essential	11	1.00	Essential	16	0.45	Essential	Y
Item39	70	1.00	Essential	11	1.00	Essential	21	0.91	Essential	Y
Item40	64	0.83	Essential	10	0.82	Essential	20	0.82	Essential	Y
Item41	68	0.94	Essential	11	1.00	Essential	20	0.82	Essential	Y

Item42	70	1.00	Essential	11	1.00	Essential	21	0.91	Essential	Y
Item43	35	0.00	Essential	7	0.27	Not Essential	13	0.18	Not Essential	N
Item44	63	0.80	Essential	11	1.00	Essential	14	0.27	Not Essential	Y
Item45	47	0.34	Essential	10	0.82	Essential	11	0.00	Not Essential	Y
Item46	60	0.71	Essential	11	1.00	Essential	14	0.27	Not Essential	Y
Item47	60	0.71	Essential	10	0.82	Essential	11	0.00	Not Essential	Y
Item48	67	0.91	Essential	11	1.00	Essential	20	0.82	Essential	Y
Item49	70	1.00	Essential	11	1.00	Essential	20	0.82	Essential	Y
Item50	58	0.66	Essential	8	0.45	Essential	13	0.18	Not Essential	M
Item51	20	-0.43	Essential	8	0.45	Essential	12	0.09	Essential	N
Item52	70	1.00	Essential	11	1.00	Essential	19	0.73	Essential	Y
Item53	62	0.77	Essential	10	0.82	Essential	17	0.55	Essential	Y
Item54	63	0.80	Essential	10	0.82	Essential	20	0.82	Essential	Y
Item55	64	0.83	Essential	11	1.00	Essential	21	0.91	Essential	M
Item56	69	0.97	Essential	11	1.00	Essential	16	0.45	Essential	Y
Item57	36	0.03	Not Essential	6	0.09	Essential	3	-0.73	Not Essential	N
Item58	66	0.89	Essential	10	0.82	Essential	18	0.64	Essential	Y
Item59	65	0.86	Essential	10	0.82	Essential	18	0.64	Essential	Y
Item60	56	0.60	Essential	8	0.45	Essential	5	-0.55	Essential	N
Item61	30	-0.14	Not Essential	5	-0.09	Not Essential	7	-0.36	Essential	N
Item62	57	0.63	Essential	9	0.64	Essential	9	-0.18	Not Essential	Y
Item63	53	0.51	Essential	10	0.82	Essential	10	-0.09	Not Essential	Y
Item64	57	0.63	Essential	11	1.00	Essential	13	0.18	Not Essential	Y
Item65	61	0.74	Essential	9	0.64	Essential	12	0.09	Not Essential	Y
Item66	44	0.26	Not Essential	5	-0.09	Not Essential	8	-0.27	Not Essential	N
Item67	30	-0.14	Not Essential	4	-0.27	Not Essential	10	-0.09	Not Essential	M
Item68	67	0.91	Essential	11	1.00	Essential	22	1.00	Essential	Y
Item69	69	0.97	Essential	10	0.82	Essential	22	1.00	Essential	Y
Item70	58	0.66	Essential	10	0.82	Essential	16	0.45	Essential	Y
Item71	54	0.54	Essential	10	0.82	Essential	12	0.09	Not Essential	M
Item72	40	0.14	Not Essential	7	0.27	Not Essential	8	-0.27	Not Essential	N
Item73	55	0.57	Essential	8	0.45	Essential	14	0.27	Not Essential	M
Item74	65	0.86	Essential	11	1.00	Essential	20	0.82	Essential	Y
Item75	68	0.94	Essential	10	0.82	Essential	22	1.00	Essential	Y
Item76	70	1.00	Essential	10	0.82	Essential	22	1.00	Essential	Y
Item77	69	0.97	Essential	10	0.82	Essential	18	0.64	Essential	Y
Item78	69	0.97	Essential	11	1.00	Essential	19	0.73	Essential	Y
Item79	69	0.97	Essential	11	1.00	Essential	21	0.91	Essential	Y
Item80	68	0.94	Essential	10	0.82	Essential	18	0.64	Essential	Y
Item81	68	0.94	Essential	10	0.82	Essential	19	0.73	Essential	Y
Item82	69	0.97	Essential	10	0.82	Essential	19	0.73	Essential	Y
Item83	62	0.77	Essential	10	0.82	Essential	12	0.09	Essential	Y
Item84	52	0.49	Essential	8	0.45	Essential	13	0.18	Not Essential	Y
Item85	58	0.66	Essential	10	0.82	Essential	14	0.27	Not Essential	Y
Item86	68	0.94	Essential	10	0.82	Essential	19	0.73	Essential	Y

Item87	68	0.94	Essential	10	0.82	Essential	22	1.00	Essential	Y
Item88	16	-0.54	Essential	9	0.64	Essential	4	-0.64	Not Essential	N
Item89	39	0.11	Not Essential	8	0.45	Essential	14	0.27	Not Essential	N
Item90	58	0.66	Essential	9	0.64	Essential	17	0.55	Essential	Y
Item91	66	0.89	Essential	11	1.00	Essential	20	0.82	Essential	Y
Item92	56	0.60	Essential	10	0.82	Essential	17	0.55	Essential	Y
Item93	46	0.31	Essential	7	0.27	Not Essential	17	0.55	Essential	N
Item94	66	0.89	Essential	11	1.00	Essential	22	1.00	Essential	Y
Item95	70	1.00	Essential	11	1.00	Essential	21	0.91	Essential	Y
Item96	37	0.06	Not Essential	9	0.64	Essential	10	-0.09	Not Essential	N
Item97	66	0.89	Essential	11	1.00	Essential	21	0.91	Essential	Y
Item98	70	1.00	Essential	11	1.00	Essential	22	1.00	Essential	Y
Item99	69	0.97	Essential	11	1.00	Essential	19	0.73	Essential	Y
Item100	13	-0.63	Essential	4	-0.27	Not Essential	5	-0.55	Not Essential	Y
Item101	56	0.60	Essential	10	0.82	Essential	13	0.18	Not Essential	N
Item102	61	0.74	Essential	10	0.82	Essential	14	0.27	Not Essential	Y
Item103	55	0.57	Essential	9	0.64	Essential	11	0.00	Not Essential	Y
Item104	63	0.80	Essential	9	0.64	Essential	17	0.55	Essential	Y
Item105	69	0.97	Essential	11	1.00	Essential	17	0.55	Essential	Y
Item106	67	0.91	Essential	10	0.82	Essential	20	0.82	Essential	Y
Item107	61	0.74	Essential	10	0.82	Essential	18	0.64	Essential	Y
Item108	60	0.71	Essential	10	0.82	Essential	16	0.45	Essential	Y
Item109	66	0.89	Essential	11	1.00	Essential	17	0.55	Essential	Y
Item110	65	0.86	Essential	11	1.00	Essential	18	0.64	Essential	Y
Item111	69	0.97	Essential	11	1.00	Essential	21	0.91	Essential	Y
Item112	64	0.83	Essential	11	1.00	Essential	19	0.73	Essential	Y
Item113	61	0.74	Essential	9	0.64	Essential	18	0.64	Essential	M
Item114	38	0.09	Not Essential	8	0.45	Essential	13	0.18	Not Essential	Y
Item115	67	0.91	Essential	11	1.00	Essential	21	0.91	Essential	Y
Item116	35	0.00	Not Essential	2	-0.64	Not Essential	14	0.27	Not Essential	N
Item117	54	0.54	Essential	10	0.82	Essential	17	0.55	Essential	Y
Item118	68	0.94	Essential	10	0.82	Essential	21	0.91	Essential	M
Item119	65	0.86	Essential	11	1.00	Essential	17	0.55	Essential	Y
Item120	54	0.54	Essential	10	0.82	Essential	16	0.45	Essential	Y
Item121	54	0.54	Essential	10	0.82	Essential	16	0.45	Essential	M
Item122	62	0.77	Essential	10	0.82	Essential	19	0.73	Essential	M
Item123	46	0.31	Essential	10	0.82	Essential	13	0.18	Not Essential	Y
Item124	41	0.17	Not Essential	5	-0.09	Not Essential	12	0.09	Not Essential	N
Item125	68	0.94	Essential	11	1.00	Essential	20	0.82	Essential	Y
Item126	64	0.83	Essential	10	0.82	Essential	17	0.55	Essential	Y
Item127	68	0.94	Essential	11	1.00	Essential	17	0.55	Essential	Y
Item128	66	0.89	Essential	11	1.00	Essential	20	0.82	Essential	Y
Item129	64	0.83	Essential	11	1.00	Essential	19	0.73	Essential	Y
Item130	63	0.80	Essential	9	0.64	Essential	19	0.73	Essential	Y
Item131	63	0.80	Essential	11	1.00	Essential	20	0.82	Essential	Y

Item132	63	0.80	Essential	11	1.00	Essential	18	0.64	Essential	Y
Item133	67	0.91	Essential	11	1.00	Essential	19	0.73	Essential	Y
Item134	67	0.91	Essential	11	1.00	Essential	15	0.36	Essential	Y
Item135	64	0.83	Essential	10	0.82	Essential	17	0.55	Essential	Y
Item136	61	0.74	Essential	9	0.64	Essential	13	0.18	Not Essential	N
Item137	57	0.63	Essential	11	1.00	Essential	16	0.45	Essential	Y
Item138	51	0.46	Essential	9	0.64	Essential	13	0.18	Not Essential	Y
Item139	60	0.71	Essential	11	1.00	Essential	16	0.45	Essential	Y
Item140	69	0.97	Essential	10	0.82	Essential	20	0.82	Essential	Y
Item141	67	0.91	Essential	11	1.00	Essential	18	0.64	Essential	Y
Item142	61	0.74	Essential	10	0.82	Essential	19	0.73	Essential	Y
Item143	64	0.83	Essential	9	0.64	Essential	19	0.73	Essential	Y
Item144	66	0.89	Essential	11	1.00	Essential	18	0.64	Essential	Y
Item145	67	0.91	Essential	9	0.64	Essential	18	0.64	Essential	Y
Item146	37	0.06	Essential	3	-0.45	Not Essential	13	0.18	Not Essential	N
Item147	67	0.91	Essential	11	1.00	Essential	21	0.91	Essential	Y
Item148	68	0.94	Essential	9	0.64	Essential	18	0.64	Essential	Y
Item149	64	0.83	Essential	10	0.82	Essential	18	0.64	Essential	Y
Item150	61	0.74	Essential	9	0.64	Essential	15	0.36	Essential	Y
Item151	69	0.97	Essential	10	0.82	Essential	20	0.82	Essential	Y
Item152	69	0.97	Essential	10	0.82	Essential	21	0.91	Essential	Y
Item153	68	0.94	Essential	10	0.82	Essential	19	0.73	Essential	Y
Item154	64	0.83	Essential	10	0.82	Essential	19	0.73	Essential	Y
Item155	61	0.74	Essential	10	0.82	Essential	19	0.73	Essential	Y
Item156	68	0.94	Essential	11	1.00	Essential	18	0.64	Essential	Y
Item157	58	0.66	Essential	10	0.82	Essential	15	0.36	Essential	Y
Item158	60	0.71	Essential	9	0.64	Essential	18	0.64	Essential	Y
Item159	47	0.34	Essential	10	0.82	Essential	17	0.55	Essential	Y
Item160	59	0.69	Essential	10	0.82	Essential	10	-0.09	Not Essential	Y
Item161	40	0.14	Not Essential	6	0.09	Not Essential	13	0.18	Not Essential	N
Item162	67	0.91	Essential	9	0.64	Essential	17	0.55	Essential	Y
Item163	60	0.71	Essential	11	1.00	Essential	16	0.45	Essential	Y
Item164	68	0.94	Essential	10	0.82	Essential	20	0.82	Essential	Y
Item165	65	0.86	Essential	11	1.00	Essential	18	0.64	Essential	Y
Item166	57	0.63	Essential	10	0.82	Essential	15	0.36	Essential	Y

Legend

CVR Threshold Value ≥ .30

Y = Yes, the item has been included in the final version of the MIC; N = No, the item has not been included in the final version of the MIC; M = Modified, the item has been modified in the latest version of the MIC.
