# Supplementary material for: Development and Validation of the Midwifery Interventions Classification for a Salutogenic Approach to Maternity Care: A Delphi Study
Source: Healthcare (Basel). 2024 Nov 8;12(22):2228. doi: 10.3390/healthcare12222228 (PMC11594468; doi:10.3390/healthcare12222228)
Supplement: Supplementary file 1 [file healthcare-12-02228-s001.zip › Table S8.pdf]

Table S8. MIC final list

	ITALIAN WORDING		ENGLISH WORDING	
	ASSISTENZA OSTETRICA DIRETTA		DIRECT MIDWIFERY CARE	
**N**	**Intervento ostetrico**	**Definizione**	**Midwifery intervention**	**Definition**
**1**	Accoglienza in una struttura sanitaria o sociale	Accogliere la donna al momento del suo ingresso in una struttura di assistenza sanitaria o sociale.	Welcome upon arrival at the facility	Welcome the birthing person upon arrival at a health or social care facility.
**2**	Accompagnamento alla nascita	Promuovere la capacità della donna di attivare le proprie competenze per affrontare travaglio e parto fornendo informazioni a sostegno di una scelta consapevole.	Antenatal education	Promote the birthing person’s ability to activate their skills to cope with labour and childbirth by providing information to support informed choice.
**3**	Ascolto attivo	Prestare grande attenzione e attribuire un significato ai messaggi verbali e non verbali della donna e del neonato.	Active listening	Pay attention to and attach meaning to verbal and nonverbal communication by the parent(s) and the newborn.
**4**	Assistenza al I stadio del travaglio	Assistere la donna dall'inizio del travaglio fino al completamento della dilatazione cervicale.	First stage of labour care	Care and support for the birthing person from the beginning of labour until complete cervical dilation.
**5**	Assistenza al II stadio del travaglio	Assistere la donna dall'inizio della dilatazione cervicale completa fino alla nascita del neonato.	Second stage of labour care	Care from complete cervical dilation until the birth of the newborn.
**6**	Assistenza al III stadio del travaglio	Assistere la donna nel periodo compreso tra la nascita del neonato e il secondamento.	Third stage of labour care	Care between the birth of the newborn and the birth of the placenta.
**7**	Assistenza al IV stadio del travaglio	Assistere la donna nelle due ore dopo il parto.	Fourth stage of labour care	Care in the first 2 hours after giving birth.
**8**	Assistenza al parto	Assistere la donna durante il parto.	Birth care	Care during childbirth.
**9**	Assistenza all'adattamento neonatale	Assistere il neonato nella fase di transizione che va dalla nascita alla vita extrauterina e alla successiva fase di stabilizzazione.	Supporting newborn adaptation	Support the newborn in the transition phase from birth to extrauterine life and the subsequent stabilization phase.
**10**	Assistenza alla fase latente del I stadio del travaglio	Assistere la donna dall'inizio della percezione di attività contrattile uterina significativa fino all'inizio del travaglio.	Early labour care	Support the birthing person from the beginning of the perception of strong uterine contractions until the start of labour.
**11**	Assistenza in gravidanza	Assistere la donna durante la gravidanza.	Antenatal care	Care during pregnancy.

**12**	Assistenza in puerperio	Assistere la donna nelle sei settimane successive al parto.	Postnatal care	Care in the 6 weeks following childbirth.
**13**	Avvio dell'allattamento al seno	Promuovere l'avvio dell'allattamento al seno e assistere la donna durante questa fase.	Facilitating breastfeeding initiation	Support with breastfeeding initiation.
**14**	Clampaggio del cordone ombelicale	Clampare il cordone ombelicale con un timing appropriato.	Cord clamping	Clamp the umbilical cord at an appropriate timing.
**15**	Condivisione di obiettivi	Collaborare con la donna per identificare gli esiti di salute attesi e stabilire il grado di priorità, allo scopo di elaborare un piano per il loro raggiungimento.	Goals sharing	Work with the pregnant/postpartum person to identify expected health outcomes, prioritise among them, and develop an action plan.
**16**	Contatto pelle a pelle (skin to skin)	Posizionare il neonato direttamente sul petto nudo della madre dopo la nascita, entrambi coperti da una coperta calda e lasciati per almeno un'ora o fino a dopo il primo pasto. Il contatto pelle a pelle può verificarsi anche ogni volta che un bambino ha bisogno di conforto o di tranquillizzarsi e può aiutare ad aumentare la produzione di latte materno.	Skin to skin	Lay the newborn directly on the birthing person's bare chest after birth, covered in a warm blanket, for at least an hour or until after the first feed. Skin-to-skin contact can also occur whenever a baby needs comforting or calming and can help boost milk supply.
**17**	Counseling	Utilizzare un processo di aiuto interattivo focalizzato sui bisogni, sui problemi o sulle sensazioni della donna, al fine di migliorare o sostenere il coping, la capacità di risolvere i problemi e le relazioni interpersonali.	Counselling	Use an interactive supportive process focused on the birthing person's needs, problems, or feelings to improve or support coping, problem-solving, and interpersonal relationships.
**18**	Counseling sui comportamenti di salute	Counseling alla donna e alla famiglia sui comportamenti di promozione della salute.	Healthy behaviour counselling	Counsel the birthing person and family on health promotion behaviours.
**19**	Counseling sui segni e sintomi	Counseling alla donna sul riconoscere segni e sintomi che richiedano consulenza ostetrica.	Signs and symptoms counselling	Counsel the birthing person on how to recognise signs and symptoms for which they should consult a healthcare professional.
**20**	Counseling sull'accudimento del neonato	Counseling alla donna e alla famiglia sulla nutrizione e sulle cure necessarie per il neonato.	Newborn care counselling	Counsel the birthing person and their family on nutrition and care of the newborn.
**21**	Counseling sull'allattamento	Counseling alla donna sull'allattamento al seno e sul chestfeeding in accordo con i dieci passi per il successo dell'allattamento al seno OMS/UNICEF.	Breastfeeding counselling	Counsel the birthing person on breast/chestfeeding, following the 10 steps to successful breastfeeding by WHO/UNICEF.
**22**	Counseling sull'eliminazione intestinale del neonato	Counseling alla donna e alla famiglia sulla fisiologia dell'eliminazione intestinale del neonato.	Newborn bowel movements counselling	Counsel the birthing person and family on the physiology of newborn bowel movements.
**23**	Counseling sull'eliminazione intestinale della donna	Counseling alla donna sulle corrette abitudini intestinali.	Counselling the birthing person on their own bowel movements	Counsel the birthing person on healthy bowel habits.
**24**	Counseling sull'eliminazione urinaria del neonato	Counseling alla donna e alla famiglia sulla fisiologia dell'eliminazione urinaria del neonato.	Newborn urinary counselling	Counsel the birthing person and family on the physiology of newborn urinary elimination.

**25**	Counseling sull'eliminazione urinaria della donna	Counseling alla donna sulle corrette abitudini urinarie.	Counselling the birthing person on their own urinary elimination	Counsel the birthing person on healthy urinary elimination habits.
**26**	Counseling sulla consapevolezza di un'assistenza alla maternità rispettosa	Counseling alle donne sul loro diritto a ricevere un'assistenza alla maternità rispettosa durante l'intero percorso nascita.	Counselling pregnant people on respectful maternity care	Counsel pregnant people on their right to respectful perinatal care.
**27**	Counseling sulla contraccezione e pianificazione di una gravidanza	Counseling alla donna e alla famiglia sulla contraccezione e sulla pianificazione di una gravidanza.	Contraception and family planning counselling	Counsel the birthing person and family on contraception and family planning.
**28**	Counseling sulla diagnosi prenatale	Counseling alla donna e alla famiglia sugli screening e sulle metodiche di diagnosi prenatale.	Prenatal diagnosis counselling	Counsel the birthing person and family on screening and prenatal diagnosis methods.
**29**	Counseling sulla nutrizione del neonato	Counseling alla donna e alla famiglia sull'alimentazione del neonato in accordo con i dieci passi per il successo dell'allattamento al seno OMS/UNICEF.	Newborn nutrition counselling	Counsel the birthing person and family on newborn nutrition, following the 10 steps to successful breastfeeding by WHO/UNICEF.
**30**	Counseling sulla nutrizione della donna	Counseling alla donna sull'alimentazione e sulla necessità di apportare modifiche alla dieta.	Nutrition counselling for the birthing person	Counsel the birthing person on their own nutrition and advise on the need to make dietary changes.
**31**	Counseling sulla salute sessuale e riproduttiva	Counseling alla donna sulla salute sessuale e riproduttiva in gravidanza e in puerperio.	Sexual and reproductive health counselling	Counsel the birthing person on sexual and reproductive health during pregnancy and the postnatal period.
**32**	Counseling sulla sicurezza del neonato	Counseling alla donna e alla famiglia sulle corrette abitudini per mantenere la sicurezza del neonato.	Counselling on newborn safety	Counsel the birthing person and family on keeping the newborn safe.
**33**	Cura del cordone ombelicale del neonato	Prevenire le complicanze del moncone ombelicale nel neonato e promuoverne la guarigione.	Umbilical cord care	Promote healing of the newborn umbilical stump and prevent complications.
**34**	Cura del perineo	Favorire il mantenimento dell'integrità perineale e alleviare il discomfort al parto.	Perineal care	Prevent perineal tearing and relieve perineal discomfort during childbirth.
**35**	Cura delle lacerazioni perineali	Prevenire le complicanze delle lacerazioni perineali e promuoverne la guarigione.	Perineal tear care	Promote the healing of perineal tears and prevent tear complications.
**36**	Esame obiettivo fisico del neonato	Valutare le condizioni fisiche generali del neonato.	Newborn physical examination	Assess the newborn's health through a physical examination.
**37**	Esame obiettivo fisico della donna	Valutare le condizioni fisiche generali della donna.	Physical examination of the birthing person	Assess the birthing person's health through a physical examination.
**38**	Gestione del dolore	Eliminare o ridurre il dolore a un livello accettabile per la donna e il neonato.	Pain management	Eliminate or reduce pain to a level acceptable to the birthing person and newborn.
**39**	Gestione dell'alimentazione del neonato	Garantire un adeguato apporto nutritivo al neonato.	Management of newborn nutrition	Ensure adequate nutrition for the newborn.

**40**	Gestione dell'alimentazione della donna	Garantire un adeguato apporto nutritivo alla donna.	Management of nutrition in pregnancy/childbirth/postpartum	Ensure adequate nutrition in the pregnancy/birth/postpartum phases.
**41**	Gestione dell'eliminazione intestinale del neonato	Garantire un'adeguata eliminazione intestinale nel neonato.	Management of newborn bowel movements	Monitor and manage the newborn's healthy bowel movements.
**42**	Gestione dell'eliminazione intestinale della donna	Garantire un'adeguata eliminazione intestinale nella donna.	Management of the birthing person's bowel movements	Monitor and support the birthing person's healthy bowel movements.
**43**	Gestione dell'eliminazione urinaria del neonato	Garantire un'adeguata eliminazione urinaria nel neonato.	Management of the newborn's micturition	Monitor the newborn's passing of urine.
**44**	Gestione dell'eliminazione urinaria della donna	Garantire un'adeguata eliminazione urinaria nella donna.	Management of the birthing person's micturition	Monitor the birthing person's passing of urine.
**45**	Gestione dell'igiene del neonato	Garantire un'adeguata igiene del neonato.	Newborn hygiene management	Ensure adequate newborn hygiene.
**46**	Gestione dell'igiene della donna	Garantire un'adeguata igiene della donna.	Hygiene management for the birthing person	Ensure adequate hygiene for the birthing person.
**47**	Gestione della regolazione della temperatura corporea	Garantire il raggiungimento e/o il mantenimento della temperatura corporea della donna e del neonato entro un intervallo di normalità.	Body temperature regulation	Ensure the birthing person's and newborn's body temperature are maintained within a normal range.
**48**	Gestione delle allergie	Identificare, trattare, prevenire le reazioni allergiche ad alimenti, farmaci, lattice, punture di insetti, mezzi di contrasto, sangue e altre sostanze.	Allergy management	Identify, treat, and prevent allergic reactions to food, drugs, latex, insect bites, contrast media, blood, and other substances.
**49**	Gestione delle alterazioni cardiotocografiche	Attuare misure conservative in risposta ad alterazioni del battito cardiaco fetale (BCF) e/o dell'attività contrattile uterina (ACU) rilevate tramite cardiotocografia.	Management of cardiotocographic changes	Implement conservative measures in response to changes in fetal heart rate (FHR) and/or uterine contractions (UC) detected by cardiotocography.
**50**	Gestione delle profilassi del neonato	Effettuare le profilassi raccomandate dalle linee guida nelle prime ore di vita del neonato.	Newborn prophylaxis	Carry out recommended prophylaxis interventions recommended by the guidelines in the first hours of newborn life.
**51**	Identificazione della persona assistita	Verificare l'identità della donna e/o del neonato assistiti.	Identification of the birthing person and/or newborn	Verify the identity of the birthing person and/or newborn.
**52**	Mantenimento dell'allattamento al seno	Promuovere il mantenimento dell'allattamento al seno e assistere la donna durante questa fase.	Facilitate breastfeeding continuation	Support with breastfeeding continuation.
**53**	Miglioramento del coping	Facilitare nella donna l'elaborazione di processi cognitivi e l'attuazione di comportamenti per la gestione dei fattori percepiti come stressanti, dei cambiamenti e delle minacce che interferiscono con il soddisfacimento delle esigenze e con lo svolgimento dei ruoli ricoperti.	Coping enhancement	Facilitate cognitive processing and the adoption of behaviours for managing factors perceived as stressful. These could include changes and threats that interfere with the satisfaction of needs and the performance of roles.

**54**	Miglioramento dell'alfabetizzazione sulla salute (health literacy)	Aiutare la donna e la famiglia ad acquisire, elaborare e comprendere le informazioni correlate alla salute e alla malattia.	Promotion of health literacy	Support the birthing person and family to acquire, process and understand information related to health and disease.
**55**	Miglioramento dell'autoefficacia (self-efficacy)	Rinforzare la fiducia della donna nella propria capacità di mettere in atto un comportamento che favorisce la salute.	Promotion of self-efficacy	Strengthen the birthing person's confidence in their ability to engage in healthy behaviours.
**56**	Miglioramento dell'autostima	Promuovere nella donna una maggiore considerazione del proprio valore.	Boosting self-confidence	Counsel the birthing person on valuing and trusting themselves.
**57**	Monitoraggio dei parametri vitali	Raccogliere e analizzare in modo sistematico i dati relativi alle funzioni cardiovascolare e respiratoria e alla temperatura corporea per identificare e prevenire le complicanze nella donna e nel neonato.	Monitoring of vital signs	Systematically collect and analyze data on cardiovascular and respiratory functions and body temperature to identify and prevent complications in the birthing person and newborn.
**58**	Monitoraggio della gravidanza a termine	Monitorare in modo costante e significativo il benessere materno-fetale al termine della gravidanza al fine di pianificare e garantire una adeguata assistenza al parto.	Monitoring of term pregnancy	Monitor parental and fetal well-being at the end of pregnancy to plan and deliver appropriate childbirth care.
**59**	Osservazione post-partum	Valutare i segni di benessere fisico ed emotivo della donna e del neonato nelle due ore successive al parto.	Early postnatal observation	Assess signs of physical and emotional well-being in the 2 hours after birth, in the birthing person and newborn.
**60**	Potenziamento della consapevolezza di sé	Assistere la donna nell'esplorare e nel comprendere i propri pensieri, sentimenti, motivazioni e comportamenti.	Promotion of self-awareness	Support the birthing person in exploring and understanding their thoughts, feelings, motivations, and behaviours.
**61**	Presenza	Essere accanto alla donna e/o al neonato, sia fisicamente sia psicologicamente, nei momenti di necessità.	Presence	Remain close to the birthing person and/or the newborn, both physically and psychologically, whenever needed.
**62**	Prevenzione dell'emorragia post-partum	Attuare misure di prevenzione dell'emorragia post-partum in accordo con le linee guida adottate.	Postpartum haemorrhage prevention	Implement post-partum haemorrhage prevention measures following the adopted guidelines.
**63**	Promozione del movimento in travaglio	Promuovere il movimento e il cambio di posizione in travaglio.	Facilitation of free movement in labour	Promote freedom of movement and changing positions during labour.
**64**	Promozione del ruolo genitoriale	Fornire informazioni sul ruolo genitoriale, supportare e coordinare i servizi disponibili per le famiglie.	Parental role promotion	Provide information on parenting, signpost, and support services available to families.
**65**	Promozione dell'attaccamento genitore-bambino (bonding)	Promuovere lo sviluppo di una relazione affettiva stabile fra bambino e genitori.	Promotion of parental-infant bonding	Support the development of a stable bond and an emotional relationship between child and parents.
**66**	Promozione dell'empowerment	Promuovere l'attivazione delle competenze della donna relative a gravidanza, parto e puerperio.	Empowerment promotion	Promote the birthing person's skills activation related to pregnancy, childbirth, and postnatal period.
**67**	Promozione della cura di sé (self-care)	Promuovere la capacità e la volontà della donna di prendersi cura di se stessa fisicamente ed emotivamente.	Promotion of self-care	Support the birthing person's willingness and ability to take care of themselves physically and emotionally.
**68**	Promozione di posizioni materne libere al parto	Promuovere la scelta materna di assumere la posizione preferita al parto.	Promotion of free movement and positions during labour and birth	Promote the birthing person's choice on what positions to use during birth.

**69**	Somministrazione di analgesici	Utilizzare farmaci per ridurre o eliminare il dolore.	Administration of analgesia	Use medication to reduce or eliminate pain.
**70**	Somministrazione di farmaci	Preparare, somministrare e valutare l'efficacia di farmaci prescritti e da banco.	Administration of medication	Prepare, administer, and assess the effectiveness of prescription and over-the-counter medication.
**71**	Sorveglianza materno-fetale in travaglio	Monitorare e documentare in modo costante e continuo il benessere materno-fetale e la progressione del travaglio.	Maternal-fetal monitoring in labour	Monitor and document maternal-fetal well-being and the progression of labour over time.
**72**	Sostegno al processo decisionale	Fornire informazioni e sostegno alla donna e alla famiglia che sta prendendo una decisione relativa all'assistenza sanitaria.	Decision-making support	Provide information and support to the birthing person and family when making decisions about healthcare.
**73**	Sostegno emozionale	Offrire rassicurazione, accettazione e incoraggiamento alla donna e/o al neonato nei momenti di stress.	Emotional support	Offer reassurance, acceptance, and encouragement to the birthing person and/or the newborn in times of stress.
**74**	Sostegno nella gestione del comportamento	Sostenere la donna nella gestione del proprio comportamento orientandolo alla promozione della salute.	Support to manage behaviour	Support the birthing person in managing her behaviour by directing her towards health promotion.
**75**	Sutura del perineo	Avvicinare i bordi di una lacerazione perineale utilizzando materiale di sutura e un ago sterili.	Perineal repair	Bring the edges of a perineal tear closer, using sterile suture material and a needle.
**76**	Triage ostetrico	Valutare in modo rapido, approfondito e sistematico il benessere materno-fetale di una donna che necessita di assistenza per determinare la priorità per una valutazione completa.	Obstetric triage	Brief, thoroughly and systematically assess the maternal-fetal well-being of a birthing person who needs assistance to determine the priority for a complete evaluation.
**77**	Valutazione del benessere emotivo	Valutare il benessere emotivo della donna in gravidanza, parto e puerperio.	Emotional well-being assessment	Assess the birthing person’s emotional well-being during pregnancy, birth, and the postnatal period.
**78**	Valutazione del benessere fetale	Valutare il benessere fetale tramite l'auscultazione e/o la registrazione del battito cardiaco fetale (BCF) e la valutazione dei movimenti attivi fetali (MAF).	Fetal well-being assessment	Assess fetal well-being through auscultation and/or fetal heart rate (FHR) recording and fetal movement (FM) assessment.
**79**	Valutazione del contesto familiare	Valutare se la donna o la famiglia hanno probabilità di incontrare difficoltà nel ruolo genitoriale e dare priorità alle strategie per prevenire questi problemi.	Consideration of the family context	Consider whether the birthing person and/or the family are likely to require support in fulfilling their parental role and signpost to appropriate services.
**80**	Valutazione dell'attività contrattile uterina	Valutare l'attività contrattile uterina (ACU) tramite palpazione e/o tocografia.	Assessment of uterine contractions	Assess uterine contractions via palpation and/or tocography.
	**ASSISTENZA OSTETRICA INDIRETTA**		**INDIRECT MIDWIFERY CARE**	
**N**	**Intervento ostetrico**	**Definizione**	**Midwifery intervention**	**Definition**
**81**	Assistenza domiciliare	Erogare cure alla donna e/o al neonato presso il domicilio.	Care at home	Provide care to the birthing person and/or the newborn in their own home.

**82**	Case management	Coordinare l'assistenza e la tutela della donna nei diversi contesti, per migliorare la qualità dell'assistenza sanitaria, garantire continuità assistenziale e raggiungere i risultati desiderati attraverso la mobilizzazione di risorse necessarie e sufficienti.	Case managament	Coordinate the care and protection of women in different contexts to improve health care quality, ensure continuity of care, and achieve the desired results by mobilising necessary and sufficient resources.
**83**	Coinvolgimento del caregiver	Coinvolgere la persona di riferimento scelta dalla donna durante gravidanza, travaglio, parto e puerperio.	Caregiver engagement	Involve and engage with the birthing's person's chosen supporter or caregiver during pregnancy, labour, childbirth, and the postnatal period.
**84**	Collaborazione con l'équipe	Collaborare e lavorare in sinergia con i componenti dell'équipe per assicurare alle donne e ai neonati un'assistenza di qualità.	Team cooperation	Collaborate and work in synergy with the members of the clinical team to ensure quality care.
**85**	Comunicazione di informazioni relative alla salute	Fornire informazioni relative alla donna e/o al neonato ad altri professionisti sanitari.	Communication of healthcare information	Provide information about the birthing person and/or the newborn to other healthcare professionals.
**86**	Consulenza assistenziale multidisciplinare	Pianificare e valutare l'assistenza alla donna e/o al neonato insieme a professionisti della salute di altre discipline.	Multidisciplinary clinical consultation	Plan and evaluate care for the birthing person and/or the newborn in collaboration with health professionals from other disciplines.
**87**	Consulenza ostetrica	Utilizzare le conoscenze di un esperto per lavorare con chi chiede aiuto per la soluzione di problemi al fine di permettere alle donne, ai neonati e alla comunità di conseguire gli obiettivi identificati.	Midwifery clinical consultation	By mobilising expert knowledge, support birthing people, newborns and the community to achieve their identified goals related to health and well-being.
**88**	Continuità assistenziale ostetrica (one to one)	Assistere la donna in gravidanza, parto e puerperio come ostetrica di riferimento o sua associata.	Midwife-led continuity of carer	Ensure that an individual midwife, or her buddy/backup, provide the majority of care to a birthing person throughout pregnancy, childbirth, and the postnatal period.
**89**	Controllo del carrello dell'emergenza	Controllare in modo sistematico e rifornire il contenuto del carrello dell'emergenza a intervalli prestabiliti.	Emergency trolley check	Systematically and regularly check and re-stock the emergency and crash trolleys.
**90**	Controllo e prevenzione delle infezioni	Ridurre al minimo l'acquisizione e la trasmissione di agenti infettivi.	Infection prevention and control	Prevent the transmission of infectious agents.
**91**	Formazione del personale sanitario su un'assistenza alla maternità rispettosa e dignitosa	Implementare corsi di formazione per il personale sanitario che promuovano conoscenza e consapevolezza e che aumentino capacità e competenze su un'assistenza alla maternità rispettosa e sulle migliori pratiche.	Training healthcare professionals on respectful perinatal care	Implement training courses for healthcare personnel that promote knowledge and increase skills on respectful perinatal care and related best practices.
**92**	Gestione dei campioni di laboratorio	Prelevare, preparare e conservare campioni per esami di laboratorio.	Management of laboratory samples	Take, prepare, and appropriately store samples for laboratory testing.
**93**	Gestione dei farmaci	Promuovere un uso sicuro ed efficace dei farmaci prescritti e da banco.	Medication management	Promote safe and effective use of prescribed and over-the-counter medications.
**94**	Gestione dell'ambiente	Organizzare l'ambiente che circonda la donna e il neonato per promuovere sicurezza, cure materne rispettose e uno stato ottimale di benessere.	Management of the environment	Organize the environment around the birthing person and the newborn to promote safety, respectful maternity care, and an optimal state of well-being.

**95**	Gestione di apparecchiature e dispositivi	Utilizzare apparecchiature e dispostivi di tipo tecnico per monitorare o sostenere le funzioni vitali della donna e/o neonato.	Management of technical equipment and devices	Use technical equipment and devices appropriately to monitor or support the vital functions of the birthing person and/or newborn.
**96**	Identificazione dei rischi	Analizzare i potenziali fattori di rischio per la donna, il neonato e la famiglia, determinare i rischi per la salute e dare la priorità alle strategie volte alla riduzione del rischio.	Risk identification	Analyse potential risk factors for the birthing person, the newborn, and the family, determine health risks, and prioritise risk-reduction strategies.
**97**	Implementazione di programmi di miglioramento della qualità dell'assistenza	Implementare programmi di miglioramento della qualità e/o audit regolari per garantire un'assistenza alla maternità rispettosa.	Implementation of healthcare quality improvement programs	Implement quality improvement programs and/or regular audits to ensure respectful perinatal care.
**98**	Implementazione di regolamenti/raccomandazioni e linee guida a supporto di un'assistenza alla maternità rispettosa	Implementare checklist standardizzate e revisionare i percorsi assistenziali ospedalieri o regionali per garantire un'assistenza alla maternità rispettosa.	Implementation of regulations/recommendations and guidelines for respectful perinatal care	Implement standardized checklists and review hospital or regional care pathways to ensure respectful perinatal care.
**99**	Interpretazione dei dati di laboratorio	Analizzare in modo critico i dati di laboratorio relativi alla donna e al neonato al fine di prendere una decisione clinica.	Interpretation of laboratory data	Critically analyze laboratory data relative to the birthing person and newborn, and use it to support clinical decision-making.
**100**	Mediazione culturale	Utilizzare in modo intenzionale strategie basate sulla competenza culturale per superare un gap o per mediare tra la cultura della donna e quella del sistema che eroga assistenza sanitaria.	Cultural mediation	Intentionally use strategies based on cultural competence to overcome a gap or to mediate between the birthing person's culture and the health system.
**101**	Miglioramento della collaborazione	Migliorare la cooperazione interdisciplinare e tra i professionisti della salute.	Enhancing interdisciplinary collaboration	Strive to achieve cooperation between healthcare professionals from different disciplines and backgrounds.
**102**	Monitoraggio della qualità	Raccogliere e analizzare in modo sistematico gli indicatori di qualità di un'organizzazione.	Quality monitoring	Systematically collect and analyse information related to the health organisation's quality indicators.
**103**	Orientamento al personale sanitario dipendente	Assistere e sostenere un neoassunto o un dipendente trasferito garantendo un orientamento pianificato verso una specifica area clinica.	Orientation of healthcare personnel	Support a newly appointed or transferred team member by orienting them to a new clinical area.
**104**	Passaggio di consegne ostetriche	Condividere con un altro gruppo ostetrico al cambio del turno di lavoro o nel trasferimento ad altro setting assistenziale le informazioni essenziali sull'assistenza alla donna e/o al neonato.	Midwifery clinical handover	Share essential and sufficient clinical information with another midwifery team upon shift change or transfer to another facility.
**105**	Personalizzazione dell'assistenza	Pianificare ed erogare un'assistenza ostetrica alla donna e al neonato focalizzata sulla loro storia e i loro bisogni individuali e sulle preferenze che esprimono.	Personalized care	Plan and deliver midwifery care focused on individuals' history, needs, and preferences.
**106**	Pianificazione dell'assistenza	Pianificare l'assistenza ostetrica in collaborazione con la donna.	Care planning	Plan midwifery care in collaboration with the birthing person.
**107**	Pianificazione della dimissione	Pianificare il rientro a domicilio o il trasferimento della donna e/o del neonato da un livello di assistenza a un altro all'interno di una struttura sanitaria oppure in un'altra struttura.	Discharge planning	Plan the discharge home or the transfer of the birthing person and/or newborn from one level of care to another within a health facility, or to another facility.

**108**	Prescrizione di un test diagnostico	Prescrivere un test diagnostico per identificare o monitorare un problema di salute.	Diagnostic test prescription	Prescribe a diagnostic test to identify or monitor a health problem.
**109**	Prescrizione di un trattamento non farmacologico	Prescrivere un trattamento non farmacologico per un problema di salute o per sostenere le funzioni dell'organismo.	Non-pharmacological treatment prescription	Prescribe non-pharmacological treatment for a health problem or to support body functions.
**110**	Prevenzione delle cadute	Adottare specifiche precauzioni per evitare le cadute nella donna e nel neonato.	Prevention of falls	Take specific precautions to avoid falls.
**111**	Promozione di un'assistenza alla maternità rispettosa (advocacy)	Promuovere e garantire un'assistenza alla maternità rispettosa.	Advocate for respectful perinatal care	Promote and ensure respectful perinatal care.
**112**	Redazione della documentazione sanitaria	Registrare nella documentazione sanitaria (cartella sanitaria ostetrica o integrata, cartacea o digitale) i dati relativi alla donna e al neonato.	Clinical record keeping	Document data related to the birthing person and newborn in their health record (midwifery or integrated health record, printed or digital).
**113**	Relazione su un evento accidentale (incident reporting)	Documentare con una relazione scritta e orale qualsiasi evento, verificatosi durante il percorso clinico-assistenziale, incongruente rispetto agli esiti attesi per la donna e/o neonato o alle attività di routine della struttura sanitaria.	Incident reporting	Document any event that occurred during the care process, which was inconsistent with expected outcomes for the birthing person and/or newborn, or with the routine functioning of the health facility.
**114**	Rooming in	Garantire alla donna e al neonato di rimanere insieme 24 ore su 24 durante la permanenza in ospedale.	Rooming-in	Ensure the birthing person and the newborn are able to stay together for 24 hours a day, while they are in the facility.
**115**	Supervisione del personale sanitario	Facilitare l'erogazione di attività assistenziale di elevata qualità da parte di altro personale sanitario.	Supervision of healthcare personnel	Facilitate the provision of high-quality care activities by other healthcare personnel, through supervision.
**116**	Supporto a chi fornisce un'assistenza alla maternità rispettosa e dignitosa	Implementare azioni e riconoscimenti per supportare chi fornisce un'assistenza alla maternità rispettosa.	Support for those providing respectful perinatal care	Implement actions and awards to support those who provide respectful perinatal care.
**117**	Sviluppo del personale sanitario	Sviluppare, mantenere e monitorare le competenze del personale.	Healthcare personnel development	Monitor, maintain, and further develop staff skills.
**118**	Tutela dei diritti della persona assistita	Tutelare i diritti della donna e del neonato relativi all'assistenza sanitaria.	Protecting the rights of birthing people and newborns	Protect the rights of the birthing person, the newborn, and their family.
**119**	Tutorato alle studentesse e agli studenti	Assistere e sostenere uno studente nelle esperienze di apprendimento.	Mentorship and supervision of students	Mentor, supervise, and support student learning experiences.
**120**	Umanizzazione dell'assistenza	Pianificare ed erogare un'assistenza ostetrica fondata sul rispetto per la dignità, l'unicità, l'individualità e l'umanità della donna e del neonato, in condizioni di lavoro adeguate e con risorse umane e materiali sufficienti.	Humanization of care	Plan and provide midwifery care based on respect for the dignity, uniqueness, individuality, and humanity of the birthing person and newborn. This requires appropriate working conditions, with sufficient human and material resources.
**121**	Valutazione dei presidi	Determinare l'efficacia di nuovi presidi o apparecchiature.	Assessment of new equipment	Determine the effectiveness of new equipment.

122	Valutazione del rischio ostetrico feto/neonatale	Analizzare i potenziali fattori di rischio ostetrico per il feto/neonato, determinare i rischi per la salute e dare la priorità alle strategie volte alla riduzione del rischio.	Risk assessment for the fetus/newborn	Analyse potential obstetric risk factors for the fetus/newborn, determine health risks, and prioritise risk-reduction strategies.
123	Valutazione del rischio ostetrico materno	Analizzare i potenziali fattori di rischio ostetrico per la donna, determinare i rischi per la salute e dare la priorità alle strategie volte alla riduzione del rischio.	Risk assessment for the birthing person	Analyse potential obstetric risk factors for the birthing person, determine health risks, and prioritise risk-reduction strategies.
	**ASSISTENZA OSTETRICA DI COMUNITÀ**		**COMMUNITY MIDWIFERY CARE**	
**N**	**Intervento ostetrico**	**Definizione**	**Midwifery intervention**	**Definition**
124	Attuazione di meccanismi di responsabilità sociale su un'assistenza alla maternità rispettosa	Implementare azioni volte a migliorare la reattività e la responsabilità nell'erogazione dei servizi.	Implementation of social accountability for respectful perinatal care	Improve the responsiveness and accountability of the service to the community, relative to respectful perinatal care.
125	Coinvolgimento di membri della comunità sull'assistenza alla maternità rispettosa	Educare i membri della comunità sull'importanza di un'assistenza alla maternità rispettosa e coinvolgerli come sostenitori per la loro comunità.	Engagement of community members with respectful perinatal care	Educate community members on the importance of respectful perinatal care and involve them as community supporters.
126	Educazione alla salute	Sviluppare e fornire informazioni ed esperienze di apprendimento per facilitare l'adattamento volontario a comportamenti che favoriscono la salute delle donne, delle famiglie e della comunità.	Health education	Develop and provide information and experiences to facilitate the voluntary adoption of healthy behaviours.
127	Gestione delle risorse economiche	Procurare risorse economiche e contribuire a deciderne l'utilizzo in modo da assicurare lo sviluppo e la continuità di programmi e servizi.	Budget management	Contribute to the management of funding streams and to decisions around budget allocation, to support the development and continuity of programmes and services.
128	Gestione delle vaccinazioni	Monitorare lo stato immunitario e facilitare l'accesso alle vaccinazioni per prevenire malattie trasmissibili.	Management of vaccinations	Monitor immune status and facilitate access to vaccinations to prevent communicable diseases.
129	Monitoraggio della politica sanitaria	Supervisionare e influenzare i regolamenti, le norme e gli standard nazionali e locali che riguardano i sistemi, la pratica ostetrica, la salute e il benessere della popolazione.	Monitoring of health policy	Keep track of and exert influence over the development of national and local regulations, norms, and standards that affect systems, midwifery practice, and ultimately the health and well-being of the population.
130	Promozione dell'attività fisica	Promuovere una regolare attività fisica per mantenere e migliorare la forma fisica e la salute.	Promotion of physical activity/exercise	Promote regular physical activity to maintain and enhance fitness and health.
131	Promozione della fisiologia della nascita	Promuovere un'assistenza alle donne e ai neonati nel rispetto della fisiologia della nascita.	Promotion of physiological processes during labour and birth	Provide care that facilitates and enhances physiological processes during childbirth.
132	Raccolta dati a fini di ricerca	Raccogliere dati per effettuare ricerche.	Data collection for research purposes	Contribute to data collection for research activities seeking to expand the evidence base related to midwifery care.
133	Screening	Rilevare i fattori di rischio o i problemi di salute utilizzando l'anamnesi, l'esame fisico e altre indagini diagnostiche.	Screening	Detect risk factors or health problems using medical history, physical examination, and other diagnostic investigations.

**134**	Sviluppo della salute della comunità	Sostenere i componenti di una comunità per identificare le preoccupazioni riguardo alla salute della comunità stessa, attivare le risorse e mettere in atto soluzioni.	Promoting health awareness at the community level	Support community members in identifying health concerns, activating resources, and implementing solutions.
**135**	Sviluppo di programmi di salute	Pianificare, attuare e valutare un insieme coordinato di attività, per migliorare il benessere o per prevenire, ridurre o eliminare uno o più problemi di salute nelle donne, nelle famiglie o nella comunità.	Development of health programmes	Plan, implement, and evaluate a coordinated set of activities to improve well-being or prevent, reduce, or eliminate one or more health problems in birthing people, families, or the community.
